# Analysis of the Antigenic and Prophylactic Properties of the *Leishmania* Translation Initiation Factors eIF2 and eIF2B in Natural and Experimental Leishmaniasis

**DOI:** 10.3389/fcimb.2018.00112

**Published:** 2018-04-05

**Authors:** Esther Garde, Laura Ramírez, Laura Corvo, José C. Solana, M. Elena Martín, Víctor M. González, Carlos Gómez-Nieto, Aldina Barral, Manoel Barral-Netto, José M. Requena, Salvador Iborra, Manuel Soto

**Affiliations:** ^1^Departamento de Biología Molecular, Facultad de Ciencias, Centro de Biología Molecular Severo Ochoa, Consejo Superior de Investigaciones Científicas (CSIC)-Universidad Autónoma de Madrid (UAM), Madrid, Spain; ^2^Departamento de Bioquímica-Investigación, Hospital Ramón y Cajal, Instituto Ramón y Cajal de Investigación Sanitaria (IRYCIS), Madrid, Spain; ^3^Parasitology Unit, LeishmanCeres Laboratory, Veterinary Faculty, University of Extremadura, Cáceres, Spain; ^4^Centro de Pesquisas Gonçalo Moniz, Fundação Oswaldo Cruz-FIOCRUZ, Salvador, Brazil; ^5^Immunobiology of Inflammation Laboratory, Department of Vascular Biology and Inflammation, Centro Nacional de Investigaciones Cardiovasculares, Madrid, Spain; ^6^Department of Immunology, School of Medicine, Universidad Complutense de Madrid, Madrid, Spain; ^7^Health Research Institute (imas12), Ciudad Universitaria, Madrid, Spain

**Keywords:** *Leishmania*, antigens, interleukin-10, visceral leishmaniasis, translation initiation factors, experimental murine models, vaccines

## Abstract

Different members of intracellular protein families are recognized by the immune system of the vertebrate host infected by parasites of the genus *Leishmania*. Here, we have analyzed the antigenic and immunogenic properties of the *Leishmania* eIF2 and eIF2B translation initiation factors. An in silico search in *Leishmania infantum* sequence databases allowed the identification of the genes encoding the α, β, and γ subunits and the α, β, and δ subunits of the putative *Leishmania* orthologs of the eukaryotic initiation factors F2 (LieIF2) or F2B (LieIF2B), respectively. The antigenicity of these factors was analyzed by ELISA using recombinant versions of the different subunits. Antibodies against the different LieIF2 and LieIF2B subunits were found in the sera from human and canine visceral leishmaniasis patients, and also in the sera from hamsters experimentally infected with *L. infantum*. In *L. infantum* (BALB/c) and *Leishmania major* (BALB/c or C57BL/6) challenged mice, a moderate humoral response against these protein factors was detected. Remarkably, these proteins elicited an IL-10 production by splenocytes derived from infected mice independently of the *Leishmania* species employed for experimental challenge. When DNA vaccines based on the expression of the LieIF2 or LieIF2B subunit encoding genes were administered in mice, an antigen-specific secretion of IFN-γ and IL-10 cytokines was observed. Furthermore, a partial protection against murine CL development due to *L. major* infection was generated in the vaccinated mice. Also, in this work we show that the LieIF2α subunit and the LieIF2Bβ and δ subunits have the capacity to stimulate IL-10 secretion by spleen cells from naïve mice. B-lymphocytes were identified as the major producers of this anti-inflammatory cytokine. Taking into account the data found in this study, it may be hypothesized that these proteins act as virulence factors implicated in the induction of humoral responses as well as in the production of the down-regulatory IL-10 cytokine, favoring a pathological outcome. Therefore, these proteins might be considered markers of disease.

## Introduction

Leishmaniases comprise a complex group of diseases caused by the infection of protozoa of the genus *Leishmania*. These parasites multiply as intracellular amastigotes within macrophages of their vertebrate hosts and as extracellular promastigotes in the gut of the insect vector (phlebotomine sand flies) (Dostálová and Volf, 2012). The parasite species as well as the immune-competence state of the host determine disease spectrum. Cutaneous leishmaniasis (CL) is the less severe form of the disease. It is caused by infection, among other species, with *Leishmania major* in the Old World and *Leishmania braziliensis* in the New World. Visceral leishmaniasis (VL) is characterized by parasite dispersion to internal organs causing a form of the disease that results deadly if treatment is not administered (Rodrigues et al., 2016). It has been estimated that there are 20,000–40,000 deaths per year due to VL in the less protected regions of the world (Alvar et al., 2012). The parasite invades the patient internal organs causing episodes of fever, weight loss, anemia, and swelling of the spleen and the liver (Herwaldt, 1999; Torres-Guerrero et al., 2017). In the Mediterranean countries, Middle-East, Asia, and South America, VL it is caused by *Leishmania infantum* [synonym *Leishmania chagasi* (Maurício et al., 2000)]. Wild canids and domestic dogs are the major reservoir of these parasites playing a central role in the transmission to humans by phlebotomine sand flies (Palatnik-de-Sousa and Day, 2011; Esch and Petersen, 2013). The infection in dogs also causes a severe form of VL complicated with different cutaneous manifestations (CanVL) (Baneth et al., 2008; Solano-Gallego et al., 2011, 2017; Abbehusen et al., 2017). For both mammalian hosts, after infection some individuals can remain asymptomatic mainly because of the induction of Th1 cellular responses and IFN-γ mediated macrophage activation for destruction of intracellular parasites. On the other hand, the symptomatic forms of the disease are associated with the generation of IL-4 mediated humoral responses against parasite antigens and an IL-10 dependent inhibition of macrophage activation (Murray, 1997; Miles et al., 2005; Baneth et al., 2008).

Visceral leishmaniasis patients possess antibodies recognizing different parasite antigens including surface molecules, some secreted factors and different intracellular proteins belonging to evolutionary conserved families that play essential cell functions. These families comprise tubulins (Abanades et al., 2012), heat shock proteins (Quijada et al., 1996, 1998), histones (Soto et al., 1999; Maalej et al., 2003), or PUF proteins (Folgueira et al., 2010). Some of these proteins families are also antigenic in CL patients (Rafati et al., 2007; Souza et al., 2013; Duarte et al., 2015). The presence of high titers of anti-*Leishmania* antibodies is thought to be linked with pathology due to the adverse effects of deposition of the immune complexes in different tissues (García-Alonso et al., 1996; Jain et al., 2000). Moreover, the presence of IgG immune complexes correlates to the down regulation of IL-12 production and the secretion of IL-10 by macrophages in mice infected with *L. major* and in human VL patients (Miles et al., 2005) depending on the density of the IgG complexes formed (Gallo et al., 2010). In addition, most of these antigens are able to induce cellular responses in CL and VL human patients or dogs affected by CanVL (Probst et al., 2001; Rafati et al., 2007; Carrillo et al., 2008; Meddeb-Garnaoui et al., 2010; Baharia et al., 2014).

During the last few years, attention has been focused on translation initiation process in *Leishmania*, since it has emerged as an important point of regulation of gene expression in these parasites (Requena, 2012). In a recent work, the parasite eIF5A has been studied at the molecular level, although its function in *Leishmania* still remains unknown (Singh et al., 2014). Studies performed with the components of the *Leishmania* eIF4F complex (eIF4A, eIF4E, and eIF4G) (Yoffe et al., 2004, 2006, 2009; Pereira et al., 2013) and poly (A)-binding proteins (PABPs) (da Costa Lima et al., 2010) have demonstrated that translation initiation in *Leishmania*, as occurs in the rest of eukaryotes, depends on the interaction with the 5′ CAP structure and poly(A) tails present in the mRNAs. Similarly, the identification of functional domains in the different subunits of *Leishmania* eIF3 complex suggests a conserved mechanism in translation initiation in *Leishmania* (Rezende et al., 2014). Little is known about the components and functions of the heterotrimeric eIF2 factor in *Leishmania*. This factor has a GTP binding activity and it is implicated in the formation of the ternary complex that recruits the Met-tRNA to the small subunit of the ribosome for translation initiation. To be functional, the participation of the multi-subunit eIF2B complex is required. This guanine exchange factor is responsible for the GDP-GTP recycling of eIF2 (Hinnebusch, 2014). In *Leishmania*, phosphorylation of the eIF2α subunit inhibits GTP recycling inducing the down regulation of global translation occurring during promastigote to amastigote differentiation (Chow et al., 2011; Cloutier et al., 2012).

Protein factors implicated in the translation process in *Leishmania* also play relevant roles regarding parasite persistence in the vertebrate host. This is the case for the *Leishmania* α-subunit of the elongation factor-1 complex (EF-1), involved in regulating the rate and fidelity of protein translation. Remarkably, this factor is able to interact and activate the macrophage tyrosine phosphatase-1 (SHP-1) resulting in macrophage deactivation and parasite survival within this cell type (Nandan et al., 2002). The recognition of some other parasite proteins implicated in translation by the host immune system has been also demonstrated. In this regard, the parasite PABPs have been found to be antigenic proteins in canine and human VL patients and humans affected by mucocutaneous leishmaniasis (MCL) due to *L. braziliensis* infection (Guerra et al., 2011; Soto et al., 2015). In addition, the *L. braziliensis* initiation factor 5a (LbeIF5A) is an antigenic protein recognized by the sera from human patients infected with *L. braziliensis* (Duarte et al., 2015). To date, the eIF4A is the most studied parasite translation initiation factor because of its relation with the host immune system. This protein seems to help the host for fighting against infection, since it was described as an inductor of Th1 mediated responses after infection in human patients (Skeiky et al., 1995) and in experimental murine models of infection (Skeiky et al., 1998). Remarkably, it is also able to activate macrophages for controlling parasite replication by the induction of pro-inflammatory cytokines (Probst et al., 1997; Koutsoni et al., 2014). Importantly, experimental vaccines based on the PABPs (Soto et al., 2015) or LeIF4A, either alone (Skeiky et al., 1998) or fused with other parasite antigens forming a recombinant poly-protein (Coler et al., 2002, 2007; Bertholet et al., 2009), have the potential of inducing protection against the infection with different *Leishmania* species. Similarly, a recombinant version of the LbeIF5a factor was able to induce protection against *L. infantum* (Duarte et al., 2016a) and *Leishmania amazonensis* (Duarte et al., 2017) challenges when administered as part of a polyprotein vaccine.

With the aim of exploring the antigenicity and prophylactic properties of other *Leishmania* proteins involved in translation, in this work we have worked with the *L. infantum* F2 (LieIF2) and F2B (LieIF2B) proteins. With this purpose, the *L. infantum* putative orthologs of the α-, β-, and γ-subunits of the eukaryotic initiation factor eIF2 and the α-, β-, and δ subunits of the eukaryotic factor eIF2B were produced as recombinant proteins and tested in ELISA using human and canine VL sera. Given that these initiation factors were found to be antigenic in natural VL, analyses were extended to a highly susceptible experimental model of VL, namely hamsters infected with *L. infantum* (Requena et al., 2000b; Carrion et al., 2006). In addition, we have analyzed the humoral and cellular responses elicited against LieIF2 and LieF2B in murine models of VL (*L. infantum*-BALB/c) and CL: susceptible (*L. major*-BALB/c) or resistant (*L. major*-C57BL/6). Finally, DNA vaccines based on both factors were constructed and assayed in BALB/c and C57BL/6 CL mouse models.

## Materials and methods

### Cloning of *L. infantum* eIF2 (LieIF2) and EiF2B (LieIF2B) factors coding regions

The DNA regions encoding the LieIF2α (LinJ.03.0960), LieIF2β (LinJ.08.0570), LieIF2γ (LinJ.09.1130), LieIF2Bα (LinJ.12.0001), LieIF2Bβ (LinJ.10.1030), and LieIF2Bδ (LinJ.27.1090) subunits were rescued from the *L. infantum* genome database (www.genedb.org) using the *Saccharomyces cerevisiae* (gene accession numbers: YJR007W, 0YPL237W, YER025W, YKR026C, YLR291C, YGR083, respectively) orthologous encoding gene sequences as queries. Genomic DNA from *L. infantum* (MCAN/ES/1996/BCN/150/MON-1) was employed as template for PCR, since no introns are present in *Leishmania* DNA (Requena, 2012). Primer sequences are available in the Supplementary Table 1. The resultant PCR products were cloned in the *BamH*I cut site of the pBluescript II SK vector (Stratagene. CA, U.S.A). DNA inserts containing the coding regions were obtained by BamHI digestion and subcloned in the same cut site of the pcDNA3.1/N-HA (pcDNA) eukaryotic expression vector (GenScript. NJ, U.S.A) for DNA vaccines, or in the same site of the pQE30 prokaryotic expression vector poly-linker (Qiagen, Hilden, Germany) for recombinant protein production.

### DNA vaccines and recombinant proteins production

For DNA vaccine, the pcDNA clones were transformed in the XL1-blue strain of *Escherichia coli* and grown. Plasmid DNA extraction was performed with the Endofree plasmid Giga Kit (Qiagen). The correct expression of the different DNA constructs was analyzed by western-blot using an anti-HA antibody (Sigma, MO, U.S.A) taking advantage of the presence of the HA tag in the N-terminal region of the recombinant proteins. Samples were obtained in COS-7 cells transfected with plasmid constructions as described in Soto et al. (2015) but using an anti-mouse IgG conjugated to horseradish peroxidase as secondary reagent (Nordic Immunological Laboratories, Tilburg, The Netherlands). *E. coli* M15 strain bacteria, transformed with the different pQE30 recombinant plasmids were employed for obtaining the *Leishmania* factors as recombinant proteins fused to an N-terminal His-tag. After over-expression of proteins in IPTG-induced cultures, the bacteria were centrifuged (5,000 × g) and total protein extracts were solubilized in binding buffer (BB; 20 mM Tris HCl pH 8.0, 0.5 M NaCl, 5 mM imidazole, 8 M urea, 1 mM 2-mercaptoethanol). For ELISA, solubilized total extracts were passed through a Ni-NTA affinity chromatography resin. Next, the column was washed in BB containing up to 20 mM imidazole. Finally, recombinant proteins were obtained from the column by passing BB containing 0.5 M imidazole. For final preparation, dialysis of the purified proteins with ELISA denaturant coating buffer was performed (EDCB: 3 M urea, 0.5 M NaCl, 5 mM imidazol, 1 mM 2-mercaptoethanol in 20 mM Tris HCl pH 8). In order to use the recombinant proteins as stimuli in cell culture assays, protein extracts were resuspended in BB. After binding to the Ni-NTA resin, interacting proteins were refolded on the affinity column as described (Shi et al., 1997). After, elution buffer was replaced by PBS using dialysis. To remove endotoxin traces, affinity chromatography polymyxin-agarose (Sigma) columns were employed. The Quantitative Chromogenic Limulus Amebocyte Assay QCL-1000 (BioWhittaker, MD, U.S.A.) was used to determine the presence of lipopolysaccharide (LPS). In all cases, proteins stocks presented <20 ng of LPS per mg of recombinant protein. Freeze-thaw total parasite extracts, namely SLA (soluble *Leishmania* antigens), were prepared as described elsewhere (Iborra et al., 2005). The Bio-Rad Protein Bradford assay (Bio-Rad, CA, U.S.A.) was employed for determination of protein concentrations.

### Mice and parasites

Female BALB/c and C57BL/6 mice (6–8 week old) were purchased from Envigo (Barcelona, Spain). Two parasite species were employed: *L. infantum* (MCAN/ES/96/BCN150) and *L. major* [clone V1 (MHOM/IL/80(Friedlin)]. Promastigotes were grown by culturing at 26°C in M3 medium (Gibco, BRL, Grand Island, NY, U.S.A) containing 10% heat inactivated fetal calf serum (FCS) and 200 U/ml penicillin and 100 μg/ml streptomycin. *L. major* metacyclic promastigotes were isolated from stationary cultures by negative selection using peanut agglutinin (Vector Laboratories, Burlingame, CA, U.S.A). For VL murine models, BALB/c mice were infected intravenously (i.v.) with 5 × 10^6^ stationary-phase promastigotes of *L. infantum* in the tail vein. For murine CL models, BALB/c or C57BL/6 mice were infected by an intradermal (i.d.) inoculation in both ears with 1 × 10^3^ metacyclic *L. major* promastigotes. In some experiments, BALB/c mice were challenged subcutaneously (s.c.) in the footpad with 1 × 10^5^ *L. major* promastigotes at the stationary-phase. All procedures were performed according to the Directive 2010/63/UE from the European Union and RD53/2103 from the Spanish Government. Procedures were approved by the next agencies: Severo Ochoa Molecular Biology Center Animal Care and Use Committee (CEEA-CBMSO 21/138), Bioethical Committee of the CSIC (under reference 100/2014), Government of the Autonomous Community of Madrid (Spain PROEX121/14).

### Sera collections

Human anonymized sera samples were randomly selected from human VL diagnosed patients stored in a serum bank (LIP-CPqGM-FIOCRUZ) built from independent studies previously conducted in Brazil in a VL endemic area (*n* = 20) (Abanades et al., 2012). Canine symptomatic VL sera (*n* = 38) were collected in the Extremadura region of Spain (previously described in Coelho et al., 2009). The sera from healthy individuals [human (*n* = 10); dogs (*n* = 13)] were also employed for comparative analysis. A collection of sera from hamster obtained from animals infected with 10^3^ (*n* = 5) or 10^4^ (*n* = 4) *L. infantum* promastigotes by the intracardiac (i.c.) route, built from a previous published study was also employed (Requena et al., 2000b). All animals (*n* = 9) presented splenomegaly and detectable parasite burdens in the spleen and liver at the end of the assay. Sera were taken before challenge and from month 1 to month 11 post-challenge. Serum samples from healthy hamsters were employed as controls (*n* = 17). To analyze the antigenicity of the translation initiation factors, murine sera collection was obtained from BALB/c mice i.v. challenged with *L. infantum* (*n* = 8) and sera from BALB/c (*n* = 8) s.c. challenged with *L. major* in the left footpad, or C57BL/6 mice (*n* = 8) i.d. challenge with the same parasite species in both ears. All sera were taken at week 8 after challenge. Sera taken from the same mice before infection were employed as negative controls. Sera from the four different mammalian species present a positive reaction against SLA (Supplementary Figure 1).

### ELISA assays

The ELISAs were performed on MaxiSorp plates (Nunc, Roskilde, Denmark). For coating, 0.2 μg per well of the recombinant proteins (in EDCB) or 1 μg per well of SLA (in PBS) were overnight incubated at 4°C. Next, four washes with PBS plus 0.5% Tween 20 (PBS-Tw) were performed. For blocking, PBS-Tw buffer supplemented with 5% non-fat milk was employed [1 h at room temperature (RT)]. For the analyses of IgG responses, human, canine, hamster, or mouse sera were assayed for 2 h at RT (1/100 dilution in the same buffer employed for blocking). Four washes with PBS-Tw were performed before incubation with secondary antibodies conjugated to horseradish peroxidase (1/2,000 dilution in the blocking solution for 1 h at RT). The following antibodies, obtained from Nordic (Nordic BioSite, Täby, Sweden) were employed: anti-dog IgG, anti-human IgG, anti-hamster IgG. Finally, after four washes in PBS-Tw the TMB ELISA substrate solution was employed for reaction developing. After incubation for 15 min in the dark, reaction was stopped by addition of 2 N H_2_SO_4_. Optical densities (O.D.) were read at 450 nm in an ELISA microplate spectrophotometer (Bio-Rad). For murine samples, the IgG1 (BALB/c and C57BL6), IgG2a (BALB/c), and IgG2c (C57BL/6) antigen-specific titers were determined by ELISA following the same procedure except that serial dilutions (1/2 dilution factor) of the sera were performed. Anti-mouse IgG1, IgG2a, and IgG2c antibodies horseradish peroxidase conjugated were employed as secondary reagents (Nordic). Sera reactivity was determined as the reciprocal end-point titer calculated as the inverse value of the highest serum dilution factor giving an absorbance value above that of the pre-immune sera.

### Analysis of the cellular immune responses

For the analysis of cytokine responses, the spleens of the mice were individually processed by mechanical homogenization in complete RPMI medium (RPMI medium supplemented with 10% heat-inactivated FCS, 20 mM L-glutamine, 200 U/ml penicillin, and 100 μg/ml streptomycin) and splenocytes passed through a cell strainer (70-μm pore size). Afterwards, cells were cultured in RPMI complete medium at 5 × 10^6^ cells per ml at 37°C in 5% CO_2_ alone or stimulated with either SLA or either LieIF2α, LieIF2β, LieIF2γ, LieIF2Bα, LieIF2Bβ, or LieIF2Bδ recombinant proteins (all of them at 12 μg/ml final concentration) for 72 h. In some experiments, a mix of the LieIF2 subunits or a mix of the LieIF2B subunits were employed (12 μg/ml final concentration; 4 μg/ml each subunit). The levels of IFN-γ, IL-10, or IL-4 were determined in culture supernatants by sandwich ELISA using monoclonal antibodies specific for mouse cytokines (capture and detection) provided in commercial kits (Pharmingen, San Diego, CA, U.S.A), following the manufacturer's instructions.

For the analysis of IL-10 production in naïve mice, spleen cultures established from BALB/c and C57BL/6 mice were cultured for 48 h in RPMI complete medium at 37°C in 5% CO_2_ with 12 μg/ml of either recombinant proteins (LieIF2α, LieIF2β, LieIF2γ, LieIF2Bα, LieIF2Bβ, or LieIF2Bδ), LPS (0.1, 1, or 10 ng/ml) or medium alone. The presence of IL-10 was determined in culture supernatants as indicated above. To identify the cells producing IL-10, equivalent cultures were prepared and stimulated. For the last 6 h BD GolgiStop protein transport inhibitor was added to the cultures. Afterwards, cells were harvested, washed twice in PBS with 1% FCS (PBS-St) and incubated with Mouse Fc Block prior to surface staining. Cells were then stained with fluorochrome-conjugated antibodies against B220 AlexaFluor 647 or CD3 AlexaFluor 647 and isotype-specific control antibodies for 30 min on ice. Cells were washed twice with PBS-St and fixed for 20 min in Cytofix/Cytoperm. Next, cells were washed in PermWash Buffer and incubated with PE-conjugated anti mouse IL-10 for 30 min at 4°C. Finally, cells were washed twice and data were collected and analyzed using a FACSCanto II Cytometer and FlowJo 10.0.7 software. All reagents and antibodies used in the cytometric assays were purchased from BD Biosciences (Franklin Lakes, NJ, U.S.A).

### Vaccination, clinical follow-up, and parasite loads

Mice (BALB/c and C57BL/6) were s.c. inoculated in the right footpad three times, 2 weeks apart, with 200 μg of the following preparations: empty pcDNA3 (200 μg), a mixture of pcDNAs containing the genes encoding LieIF2 subunits (LieIF2α, LieIF2β, and LieIF2γ; 67 μg each) or a mixture of of pcDNAs containing the genes encoding LieIF2B subunits (LieIF2Bα, LieIF2Bβ, and LieIF2Bδ, 67 μg each) prepared in PBS. As an additional control, a group of mice received the same volume (30 μl) of the vaccine diluent. For both mouse strains, animals (*n* = 8 per group) were euthanized at the time of infection to analyze the immune response elicited by vaccination. Parasite challenge was carried out at the fourth week after vaccination s.c. in the left footpad of the BALB/c mice or i.d. in the ear dermis of both BALB/c and C57BL/6 mouse strains with the doses depicted above (section Mice and Parasites). For the analysis of the clinical signs of infection, the footpad swelling (thickness of the infected left footpad minus thickness of the right footpad) or the diameter of the ear lesions was weekly measured with a metric caliper. BALB/c mice s.c. infected in the footpad were euthanized at week 8 post-challenge (*n* = 8). The BALB/c mice i.d. infected in the ear dermis were euthanized at week 5 (*n* = 5) and at week 8 (*n* = 5), whereas C57BL/6 mice were euthanized at week 5 (*n* = 5) and at week 9 post-challenge (*n* = 5). For parasite load determination, the popliteal lymph nodes (LNs) draining the footpad (for s.c. infected animals), the ears, the retromandibular draining LNs and the spleen of each mouse were individually taken and mechanically homogenized in Schneider's complete medium (Schneider's medium supplemented with 20% heat-inactivated FCS, 200 U/ml penicillin, and 100 μg/ml streptomycin). Ear sheets were previously digested for 2 h at 37°C in Dulbecco's modified Eagle medium containing Liberase TL enzyme (50 μg/ml; Roche Diagnostics, Mannheim, Germany). Samples were serially diluted (1/3) in Schneider's complete medium and plated on triplicates in a 96-well plates. The number of viable parasites was calculated from the highest dilution at which promastigotes could be grown with up to 10 days of incubation at 26°C (Buffet et al., 1995).

### Statistical analysis

Statistical analyses were performed using the Graph-Pad Prism Program. A D'Agostino and Pearson test was employed to analyze the Gaussian distribution of the samples when *n* ≥ 8. The ELISAs cut-off values were calculated by comparison of the reactivity values from infected and healthy groups using a Receiver Operating Characteristic (ROC) analysis and defined as the lowest O.D. value with a 100% of specificity. The Mann-Whitney or the Kruskal-Wallis (followed by Dunn's multiple comparison analysis) tests were employed for analyzing two or more samples, respectively. Significant differences was indicated as ^*^, ^+^, ^x^ for *P***-**value lower than 0.05, ^**^, ^++^, ^xx^ for *P*-value lower than 0.01 or ^****^, ^+++^, ^xxx^ for *P* value lower than 0.001.

## Results

### Identification of *Leishmania infantum* eIF2 and eIF2B factors

The genes encoding *L. infantum* eIF2 (LieIF2) and eIF2B (LieIF2B) factors were identified in the sequence database of *L. infantum* (www.genedb.org) by BLAST searches employing the *S. cerevisiae* orthologs sequences. We retrieved the entries annotated as the putative α, β, and γ subunits of the LieIF2 and α, β, and δ subunits of the LieIF2B complexes. As it is shown in Table 1, the high degree of conservation found for these proteins among different *Leishmania* spp. decreases when they are compared to their putative orthologs in yeast (the species selected for *in silico* search) and humans (one of the mammalian hosts of the parasite). To get additional evidence about the identity of the rescued proteins, an *in silico* search for conserved domains was performed (http://www.ebi.ac.uk/interpro/). It was found that the *L. infantum* subunits of both complexes possess equivalent structural domains to those present in the human ones, including the GTP binding domain located at the amino-terminal region of the eIF2-γ subunit (Pain, 1996).

**Table 1:** Amino acid sequence comparison analysis.

***Leishmania infantum***	***Leishmania major***	***Saccharomyces cerevisiae***	***Homo sapiens***
LieIF2α	98.6 (99.3)	34.8 (58.4)	31.0 (51.1)
LieIF2β	99.1 (99.7)	25.6 (45.8)	25.8 (41.8)
LieIF2γ	99.6 (99.6)	50.3 (69.9)	53.5 (72.0)
LieIF2Bα	97.7 (98.7)	24.1 (43.3)	27.9 (49.0)
LieIF2Bβ	97.4 (97.9)	23.2 (36.6)	24.7 (37.4)
LieIF2Bδ	96.2 (97.7)	23.3 (40.9)	25.0 (41.1)

*The percentages of identity (similarity) are shown, obtained using the “water” tool of the EMBOSS suite of bioinformatics. L. infantum and yeast gene accession numbers are included in the Materials and Methods section. L. major genes accession numbers are: LmjF.03.0980, LmjF.08.0550, LmjF.09.1070LmjF.12.0010, LmjF.10.0950, LmjF.27.1210. Human NCBI reference sequence entries are: NP_004085.1, NP_003899.2, NP_001406.1, NP_001405.1, NP_055054.1, NP_001029288.1*.

### Antigenicity of *Leishmania infantum* eIF2 and eIF2B factors in natural and experimental VL

We produced the six indicated subunits of the LieIF2 and LieIF2B translation factors in *E. coli* and the resulting His-tagged recombinant proteins were purified by affinity chromatography. Firstly, we analyzed their antigenicity by ELISA using different sera collections. The recognition of sera collected from VL Brazilian human patients ranged from 85.0% for LieIF2α to 20.0% for LieIF2Bα. Data were summarized in Table 2 and the details regarding the sera reactivity, determination of the cut-off values and the statistical analysis were included in Supplementary Figure 2A. The antigenicity of the subunits in the canine form of the disease was also demonstrated, ranging the percentages of positive samples between 73.7% for LieIF2γ and LieIF2Bβ to 57.9% for LieIF2α and LieIF2Bα (Table 2; Supplementary Figure 2B).

**Table 2:** Percentages of sera positive for *Leishmania* eIF2 and eIF2B subunits.

**Protein**	**Human VL**	**Canine VL**	**Hamster VL**
LieIF2α	85.0	57.9	100
LieIF2β	45.0	65.8	100
LieIF2γ	60.0	73.7	100
LieIF2Bα	20.0	57.9	100
LieIF2Bβ	50.0	73.7	100
LieIF2Bδ	80.0	71.1	100

*Human (n = 20), canine (n = 38), and hamster (n = 9) sera were assayed by ELISA against the different subunits, obtained as recombinant proteins*.

**Figure 1:**
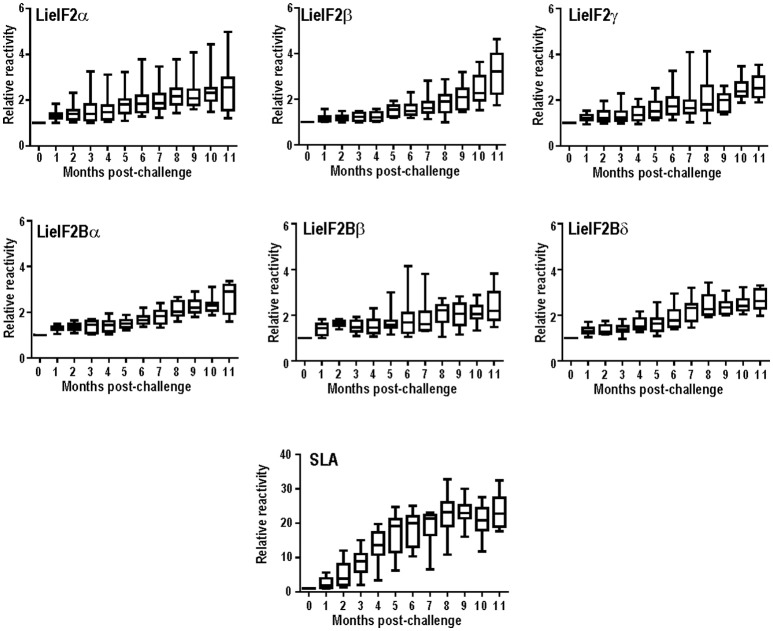
Time course of anti-leishmanial humoral response in infected hamsters. The presence of antibodies against the subunits of the LieIF2 factor (α, β, and γ), the LieIF2B factor (α, β, and δ) or SLA was determined by ELISA using the sera from nine hamster infected by the intracardiac route with *L. infantum* promastigotes. Sera were collected before challenge and monthly after infection until month 11th. Whisker (min to max) plots were employed to show the relative absorbance of the sera (= absorbance values of a given serum obtained from an infected hamster divided by the absorbance value of the pre-immune sera from the same animal).

Next, we extended the studies to an experimental model of the VL disease, namely hamster experimentally infected with *L. infantum*. Eleven months after challenge, all the infected animals had circulating antibodies against the subunits of the LieIF2 and LieIF2B complexes (Table 2; Supplementary Figure 2C). Additionally, in *L. infantum*-infected hamsters, we studied the time course of appearance of anti-LieIF2 and anti-LieIF2B antibodies in parallel to anti-SLA along the infection. With this purpose, hamster sera samples were collected monthly after *L. infantum* challenge and analyzed by ELISA. A continuous increase in the reactivity against either LieIF2 or LieIF2B subunits was observed from the first month post-infection reaching the highest reactivity value at the end of the assay, following a similar kinetics to the SLA recognition (Figure 1).

### Immune responses elicited against the LieIF2 and LieIF2B subunits in murine models of VL and CL

To further analyze the antigenic properties of the LieIF2 and LieIF2B translation factors we take advantage of the high amino acid sequence identity shared between the orthologous subunits from *L. infantum* and *L. major* (Table 1). We studied the humoral and cellular responses elicited against the recombinant versions of these subunits using sera samples as well as spleen cells from BALB/c mice infected either with *L. infantum* (VL murine model) or with *L. major* (CL susceptible model). Samples from C57BL/6 mice infected with *L. major* were also analyzed (CL resistant model). A moderate humoral response was found against the LieIF2 and LieIF2B factors in these animals. All the subunits were recognized by the sera from infected mice with percentages ranging from 62.5 to 100% (Table 3 and Supplementary Figures 3A–C).

**Table 3:** Percentages of murine sera positive for *Leishmania* eIF2 and eIF2B subunits.

**Protein**	**VL**	**CL susceptible**	**CL resistant**
LieIF2α	87.5	75	87.5
LieIF2β	87.5	87.5	100
LieIF2γ	100	100	75
LieIF2Bα	87.5	100	62.5
LieIF2Bβ	87.5	87.5	87.5
LieIF2Bδ	62.5	100	100

*Sera (n = 8 per group) from BALB/c mice infected with L. infantum (VL) or L. major (CL susceptible) and C57BL/6 infected with L. major were assayed by ELISA against the different subunits, obtained as recombinant proteins*.

**Figure 2:**
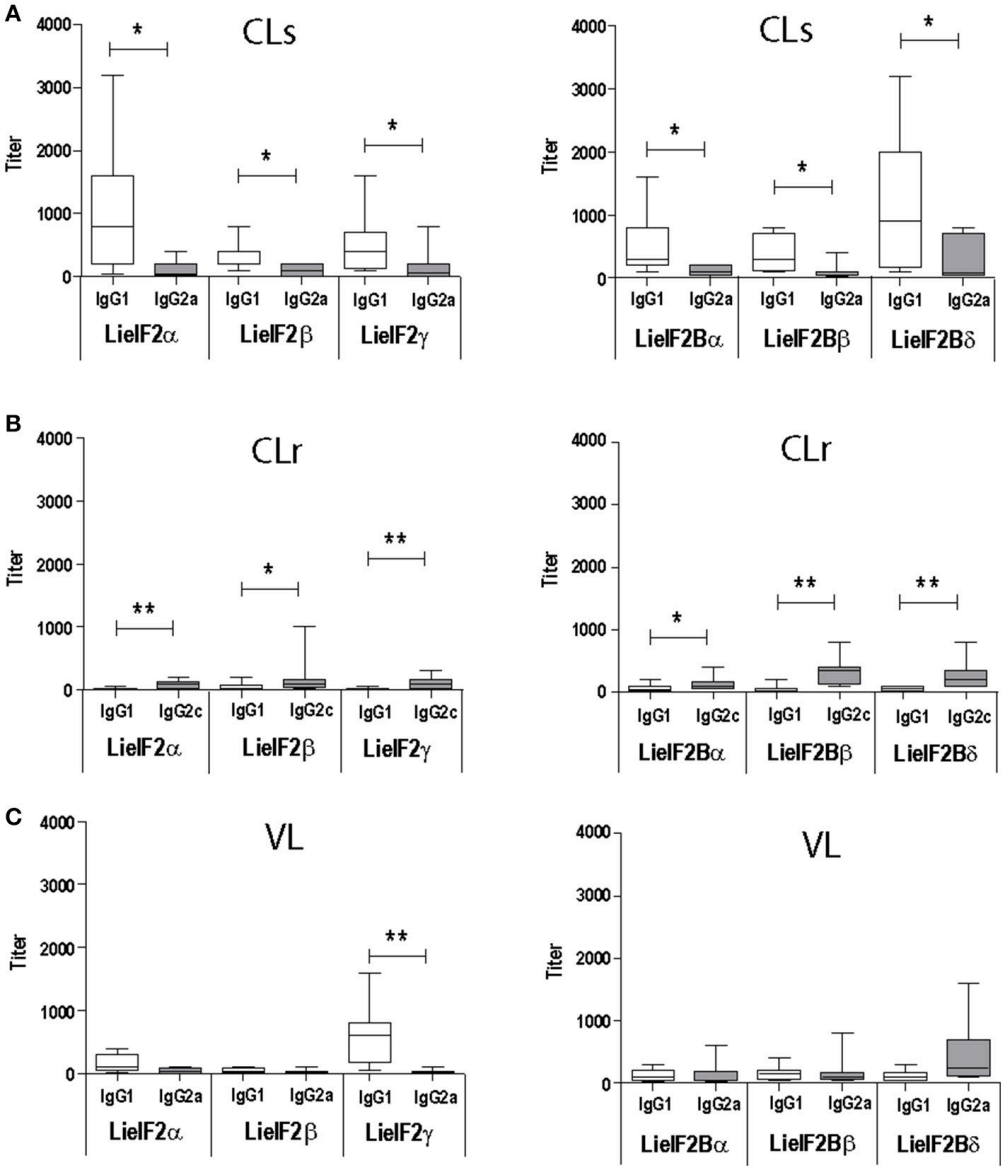
Analysis of the humoral response elicited against LieIF2 and LieIF2B in murine models of leishmaniasis. Mice (*n* = 8) per group were experimentally challenged with *L. major* [susceptible BALB/c mice, CLs **(A)**; resistant C57BL/6 mice, CLr **(B)**] or with *L. infantum* [BALB/c mice, VL**(C)**]. The IgG1 (BALB/c and C57BL/6), IgG2a (BALB/c), or IgG2c (C57BL/6) titers against the indicated LieIF2 (left panels) and LieIF2B (right panels) subunits were determined by ELISA at week 8 post-challenge. Asterisks show the statistical differences between the indicated subclasses (Mann–Whitney test). Results in each panel are representative of two independent experiments.

As occurs for anti-SLA antibodies (Supplementary Figure 4A) the immunoglobulins recognizing the six subunits were predominantly of the IgG1 subclass in *L. major* infected BALB/c mice (Figure 2A). In C57BL/6 mice a weak IgG2c predominant response against the different subunits was observed (Figure 2B). A mixed IgG1/IgG2a response against all the subunits was observed in *L. infantum* infected BALB/c mice with the exception of LieIF2γ that was predominantly recognized by IgG1 antibodies (Figure 2C).

**Figure 3:**
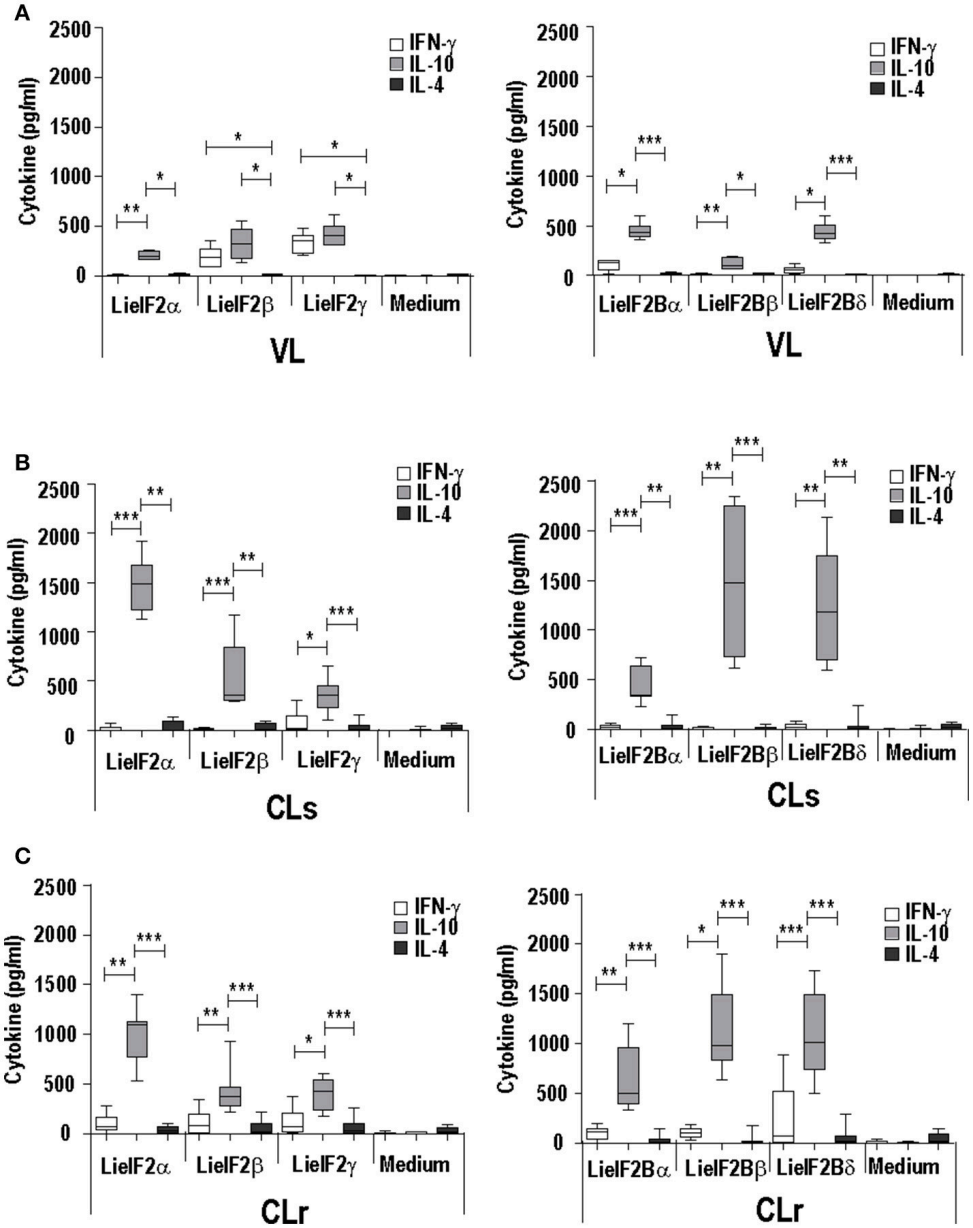
Analysis of the cellular response elicited against LieIF2 and LieIF2B in murine models of leishmaniasis. Mice (*n* = 8) per group were experimentally challenged with *L. infantum* (BALB/c mice, VL) **(A)** or with *L. major* [susceptible BALB/c mice, CLs **(B)**; resistent C57BL/6 mice, CLr **(C)**]. At week 8 post-challenge spleen cells were prepared and cultured in the absence (Medium) or in the presence of the indicated LieIF2 and LieIF2B subunits. The presence of IFN-γ, IL-10, and IL-4 cytokines in culture supernatants was measured by sandwich ELISA. The results are represented as Whisker (min to max) plots. Asterisks mark the statistical differences among the three assayed cytokines (Kruskal–Wallis test). Results in each panel are representative of two independent experiments.

The analysis of the cytokine secretion after *in vitro* stimulation of spleen cells from infected mice with the recombinant subunits revealed a predominant IL-10 mediated response in all experimental murine models. For the three LieIF2 (Figures 3A–C) and LieIF2B (Figures 3A–C) subunits, the levels of IL-10 in culture supernatants were significantly higher than the other two assayed cytokines (IFN-γ and IL-4), with the exceptions of LieIF2β and LieIF2γ factors in the VL model (Figure 3A). The levels of IFN-γ detected in the supernatants of spleen cell cultures stimulated with the recombinant factors were always lower than IL-10 amounts (Figures 3A–C) contrasting with the parasite specific IFN-γ predominant response observed when the same cells were stimulated with SLA especially in VL and CL resistant models (Supplementary Figure 4B).

### Administration of the LieIF2 and LieIF2B subunits as DNA vaccines induces a partial protection against CL in both susceptible and resistant murine models

**Figure 4:**
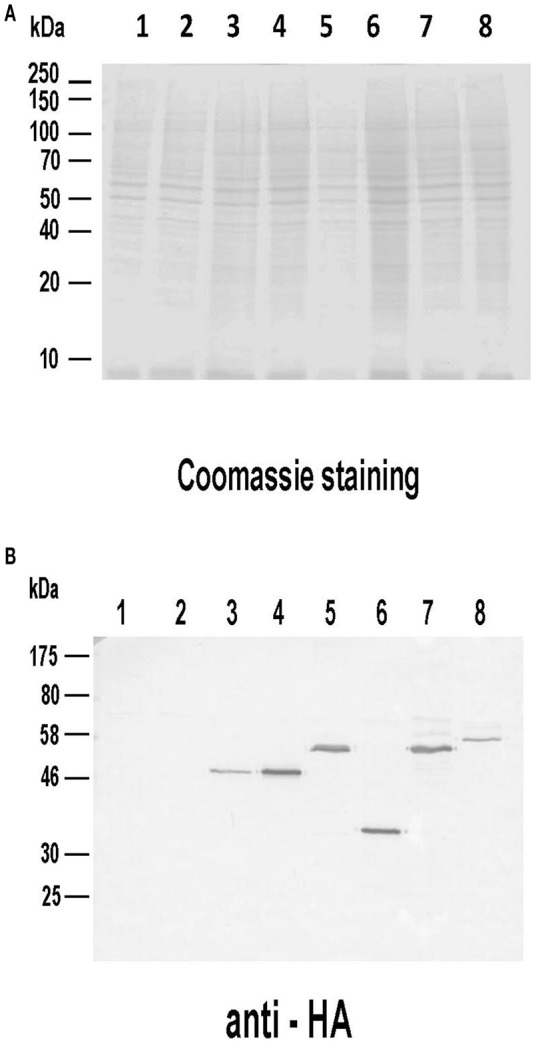
Analysis of the expression of the DNA vaccines coding for the LieIF2 and LieIF2B subunits. Untransfected COS7 (lane 1) as well as cells transfected with pcDNA-HA (lane 2), pcDNA-HA-LieIF2α (lane 3), pcDNA-HA-LieIF2β (lane 4), pcDNA-HA-LieIF2γ (lane 5), pcDNA-HA-LieIF2Bα (lane 6), pcDNA-HA-LieIF2Bβ (lane 7), and pcDNA-HA-LieIF2Bδ (lane 8) for 72 h cells were harvested, lysed and separated on a 10% SDS-PAGE gel and stained with Coomassie blue **(A)**. A western blot performed with an equivalent gel was incubated sequentially with an anti-HA antibody made in mouse and an anti-mouse IgG antibody labeled with horseradish peroxidase and revealed with 4-chloro-1-naphthol **(B)**.

Given the remarkable antigenicity of these *Leishmania* proteins in different hosts, we analyzed their immunogenicity when administered as genetic vaccines (injection of naked DNA eukaryotic expression plasmids). The correct expression of the different plasmid constructs was demonstrated by western blot using total protein extracts from COS cells transfected with the plasmid collection (Figure 4A). Proteins bands of expected sizes were recognized by an antibody specific for the HA tag located in the N-terminal of the recombinant *Leishmania* subunits produced in the mammalian cells (Figure 4B).

**Figure 5:**
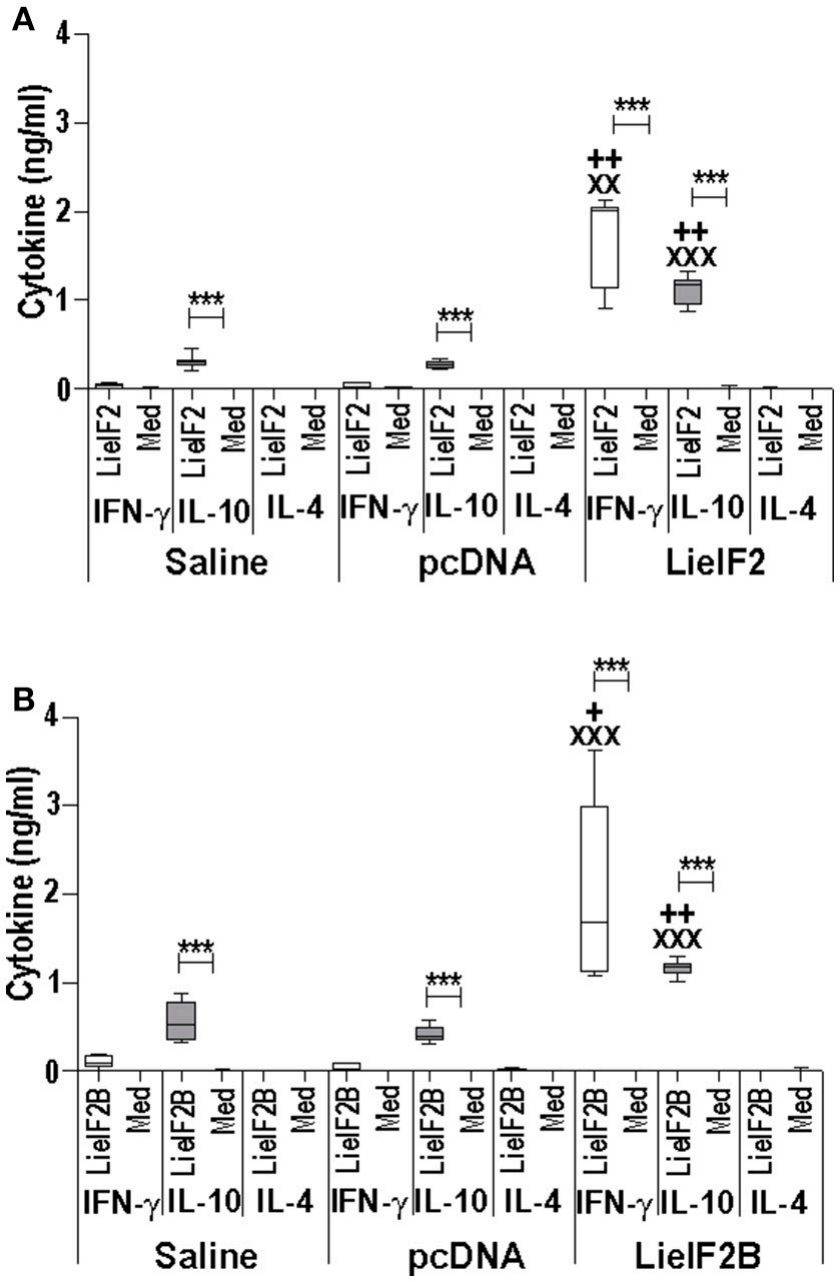
Cytokine response induced by LieIF2 and LieIF2B-based genetic vaccination in BALB/c mice. Three groups of BALB/c mice (*n* = 8 per group) were inoculated with PBS (Saline group), with the pcDNA non-recombinant vector (pcDNA group) of with a mixture of the pcDNA-LieIF2α + pcDNA-LiF2β + pcDNA-LieIF2γ plasmids (LieIF2 group) or alternatively, with a mixture of the pcDNA-LieIF2Bα + pcDNA-LiF2Bβ + pcDNA-LieIF2δ plasmids (LieIF2B group) three times, 2 weeks apart. Four weeks after the last dose, spleen cells from mice of the saline, pcDNA and LieIF2 or LieIF2B vaccinated groups were extracted and cultured in the presence or in the absence (Med), and a mixture of the LieIF2 subunits (LieIF2α, LieIF2β, and LieIF2γ) for LieIF2 vaccinated mice **(A)** or a mixture of the LieIF2B subunits (LieIF2Bα, LieIF2Bβ, and LieIF2δ) for LieIF2B vaccinated animals **(B)**. Graphs show the level of the indicated cytokines in the cell culture supernatant determined by sandwich ELISA. Results are presented as Whisker (min to max) plots. Asterisks show the statistical differences between the level of cytokines in the supernatants of the stimulated and the non -stimulated cultures (Mann–Whitney test), whereas the ^+^ or the ^X^ symbols show the statistical differences among saline and vaccinated mice or pcDNA and vaccinated mice, respectively (Kruskal–Wallis test).

**Figure 6:**
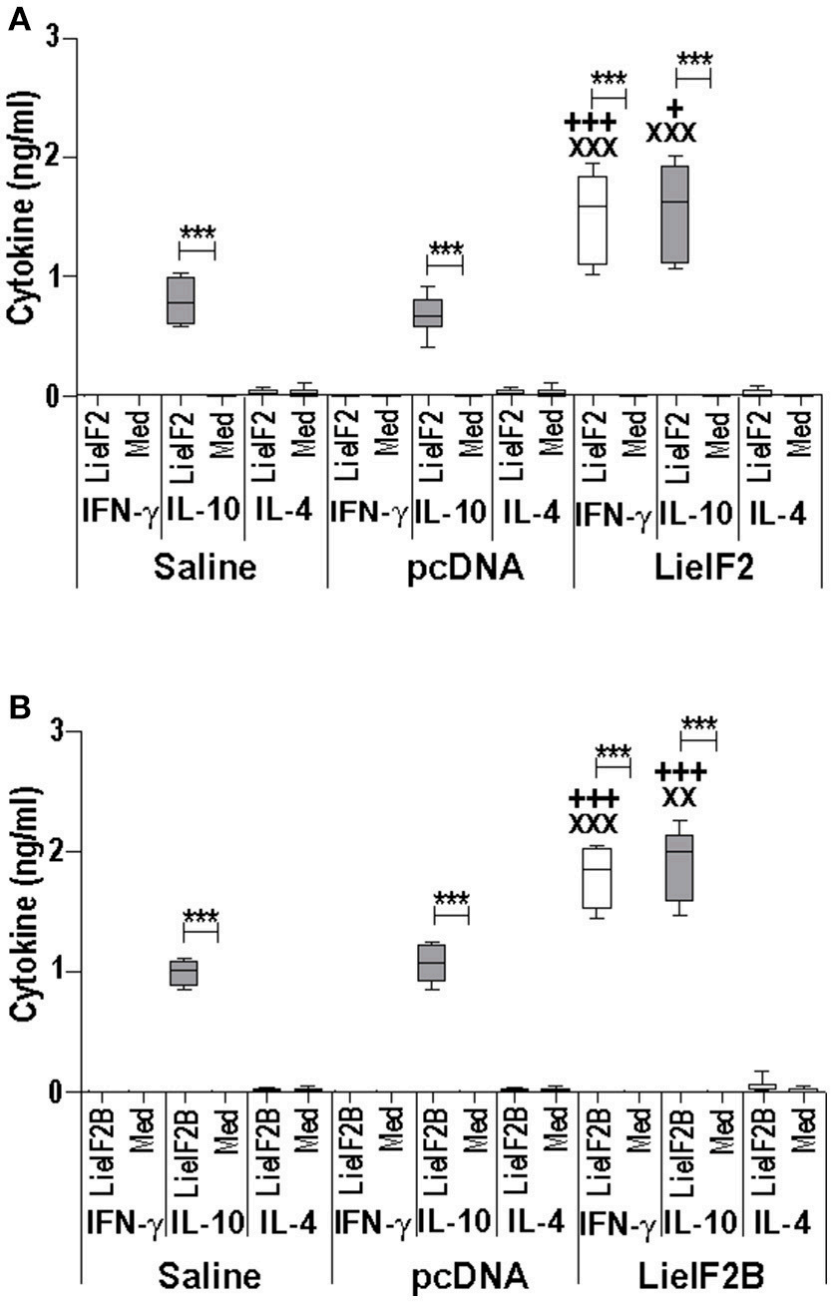
Cytokine response induced by LieIF2 and LieIF2B-based genetic vaccination in C57BL/6 mice. Three groups of C57BL/6 mice (*n* = 8 per group) were inoculated with PBS (Saline group), with de pcDNA non-recombinant vector (pcDNA group) of with a mixture of the pcDNA-LieIF2α + pcDNA-LiF2β + pcDNA-LieIF2γ plasmids (LieIF2 group) or alternatively, with a mixture of the pcDNA-LieIF2Bα + pcDNA-LiF2Bβ + pcDNA-LieIF2δ plasmids (LieIF2B group) three times, 2 weeks apart. Four weeks after the last dose, spleen cells from mice of the saline, pcDNA and LieIF2 or LieIF2B vaccinated groups were extracted and cultured in the presence, or in the absence (Med), of a mixture of the LieIF2 subunits (LieIF2α, LieIF2β, and LieIF2γ) for LieIF2 vaccinated mice **(A)** or a mixture of the LieIF2B subunits (LieIF2Bα, LieIF2Bβ, and LieIF2δ) for LieIF2B vaccinated animals **(B)**. Graphs show the level of the indicated cytokines in the cell culture supernatant determined by sandwich ELISA. Results are presented, as Whisker (min to max) plots. Asterisks show the statistical differences between the level of cytokines in the supernatants of the stimulated and the non-stimulated cultures (Mann–Whitney test), whereas the ^+^ or the ^X^ symbols show the statistical differences among saline and vaccinated mice or pcDNA and vaccinated mice, respectively (Kruskal–Wallis test).

Two different DNA vaccines were assayed: the LieIF2 vaccine, composed by the same amounts of plasmids encoding the LieIF2α, LieIF2β, and LieIF2γ subunits, and the LieIF2B vaccine, containing the same amounts of the plasmids encoding the LieIF2Bα, LieIF2Bβ, and LieIF2Bδ factors. To characterize the immune response elicited by the vaccines, spleen cells from vaccinated and control mice (immunized with the saline excipient or the non-recombinant plasmid) were cultured in the presence or in the absence of a mixture of the subunits composing each one of the vaccines. Administration of the genetic vaccines either in BALB/c (Figures 5A,B) or in C57BL/6 mice (Figures 6A,B) resulted in the induction of both IFN-γ and IL-10 mediated responses against LieIF2 or LiF2B subunits. Remarkably, when cells of the two control mouse groups were stimulated with a mixture of either the LieIF2 or LieIF2B factors a specific IL-10 response was observed, although of lesser magnitude regarding vaccinated groups (Figures 5, 6). This comportment will be analyzed in more detail in the last subsection of the results.

**Figure 7:**
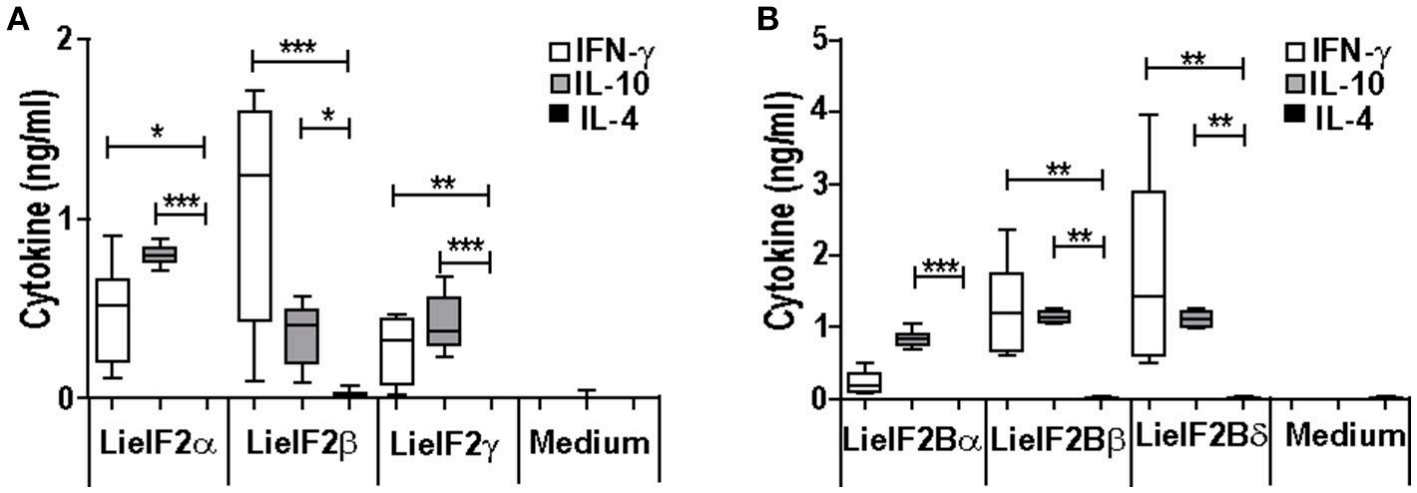
Cytokine response against LieIF2 and LieIF2B subunits in vaccinated BALB/c mice. Spleen cells cultures established from BALB/c mice vaccinated with LieIF2 and LieIF2B genetic vaccines as indicated in the legend of Figure 5 were stimulated with the single LieIF2 subunits (LieIF2α, LieIF2β, and LieIF2γ) for LieIF2 vaccinated mice **(A)** or the single LieIF2B subunits (LieIF2Bα, LieIF2Bβ, and LieIF2δ) for LieIF2B vaccinated animals **(B)** or grown in medium alone. Graphs show the level of the indicated cytokines in the cell culture supernatant determined by sandwich ELISA. Results are presented, as Whisker (min to max) plots. Asterisks mark the statistical differences among the levels found in the culture supernatants of the three assayed cytokines (Kruskal–Wallis test). Results in each panel are representative of two independent experiments.

**Figure 8:**
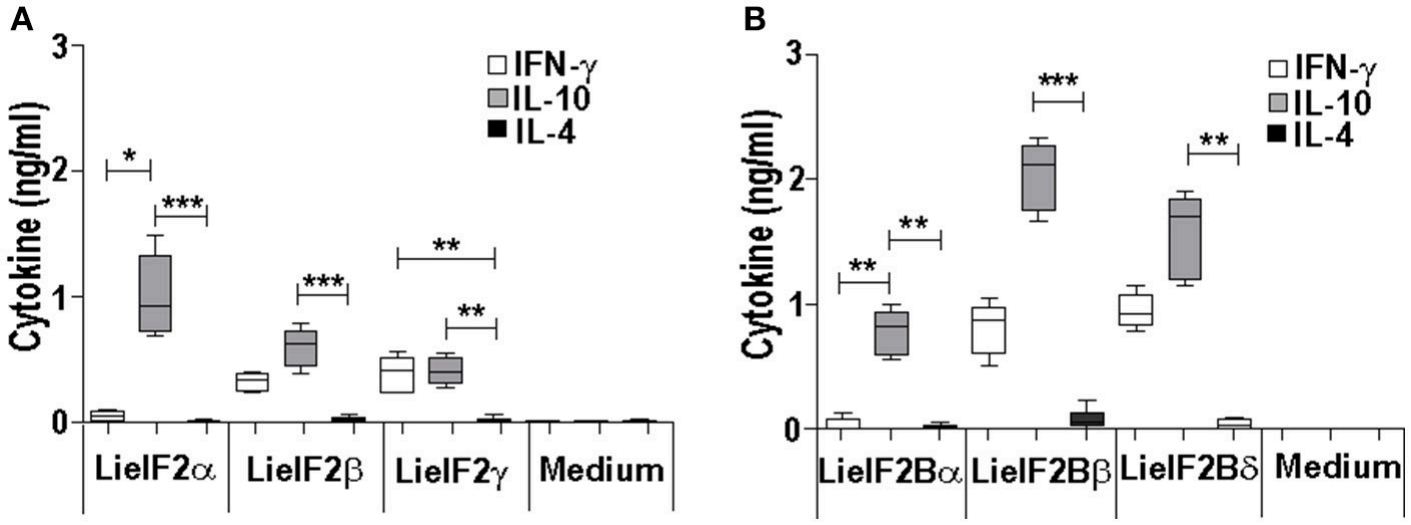
Cytokine response against LieIF2 and LieIF2B subunits in vaccinated C57BL/6 mice. Spleen cells cultures established from C57BL/6 mice vaccinated with LieIF2 and LieIF2B genetic vaccines as indicated in the legend of Figure 6 were stimulated with the single LieIF2 subunits (LieIF2α, LieIF2β, and LieIF2γ) for LieIF2 vaccinated mice **(A)** or the single LieIF2B subunits (LieIF2Bα, LieIF2Bβ, and LieIF2δ) for LieIF2B vaccinated animals **(B)** or grown in medium alone. Graphs show the level of the indicated cytokines in the cell culture supernatant determined by sandwich ELISA. Results are presented, as Whisker (min to max) plots. Asterisks mark the statistical differences among the levels found in the culture supernatants of the three assayed cytokines (Kruskal–Wallis test). Results in each panel are representative of two independent experiments.

Additional immune-stimulation assays were done using individual recombinant subunits instead of mixtures to *in vitro* stimulate spleen cells from vaccinated mice. For BALB/c mice, antigen-specific production of both IFN-γ and IL-10 was elicited by all three LieF2 (Figure 7A) and LieIF2B (Figure 7B) subunits, since we did not observe significant differences in the levels of both cytokines for any of the subunits. However, we detected a slight tendency to generate higher levels of IL-10 by LieIF2α, LieIF2γ (Figure 7A), and LieIF2Bα (Figure 7B) subunits, whereas LieIF2β (Figure 7A) and LieIF2Bβ or LieIF2Bδ (Figure 7B) culture supernatants presented a slight predominance of IFN-γ. In splenocytes from vaccinated C57BL/6 mice, an antigen-specific production of IFN-γ and IL-10 was also observed. In this mice strain, a predominant IL-10 mediated response for all the subunits of the LieIF2 (Figure 8A) and LieIF2B (Figure 8B) factors was observed. This was especially marked when the LieIF2α (Figure 8A) and the LieIF2Bα (Figure 8B) subunits were employed as stimuli. In these cultures the level of IL-10 was statistically incremented with regard to the other studied cytokines. All the subunits stimulated the production of very low levels of IL-4 in both mouse strains.

**Figure 9:**
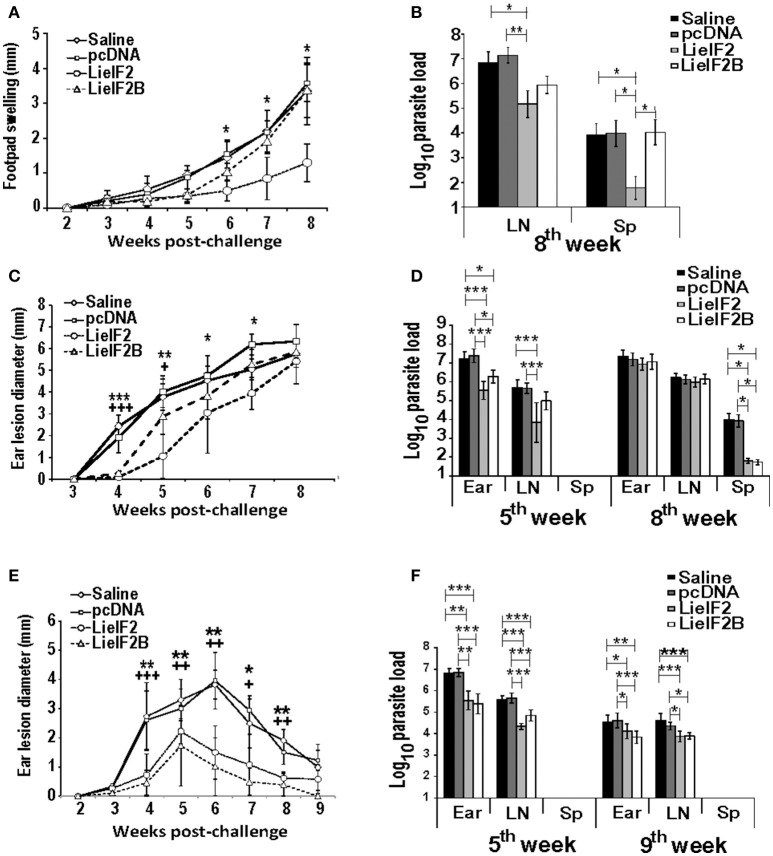
Course of *L. major* infection in vaccinated mice. BALB/c (*n* = 18 per group) or C57BL/6 (*n* = 10 per group) were inoculated with saline, with pcDNA, with the LieIF2 or with the LieIF2B genetic vaccines three times 2 weeks apart. Four weeks after the last inoculation mice were challenge with *L. major*, following the next scheme: 5 × 10^5^ stationary phase promastigotes in the footpad (BALB/c, *n* = 8 per group) or 1 × 10^3^ metacyclic promastigotes in the dermis of both ears (BALB/c and C57BL/6, *n* = 10 per group). In **(A)** the mean ± *SD* of the difference of thickness between the infected and the uninfected footpad (*n* = 8 mouse per group) is represented. The asterisks symbolize the statistically decrease of footpad swelling of the LieIF2 vaccinated group with respect saline or pcDNA groups (Kruskal–Wallis test). In **(C)** (BALB/c) and **(E)** (C57BL/6), the ear lesion diameter (mean ± *SD*) is shown (*n* = 20 ears from week 2 to week 5 post-challenge and *n* = 10 ears from week 6 to the end of the assay). The statistically significant decrease of ear lesion found in LieIF2 (^*^ asterisks) or in LieIF2B (+ symbol) vaccinated mice with respect saline of pcDNA group is shown (Kruskal-Wallis test). In the three panels, and for simplicity, the lower value of significance found when data from vaccinated were compared to saline and pcDNA control data is indicated in the graph. The number of viable parasites in the left popliteal LNs and spleens (BALB/c mice infected in the footpad; **B**), or the ears, the retromandibular LNs and the spleens in BALB/c **(D)** or C57BL/6 **(F)** mice challenged in the ear dermis were individually determined by limiting dilution at the indicated weeks post-challenge. Mean ± *SD* of the log10 of the parasite burdens in the complete organs is shown. Asterisks represent the significant differences between the indicated groups (Kruskal–Wallis test). In **(B)**, biological samples from eight animals per group were employed. In **(D,F)**, samples from five animals were employed at the indicated times post-challenge. All the samples were processed individually.

Finally, we tested the immune-prophylactic properties of these DNA vaccines by challenging control and vaccinated mice with *L. major*. For BALB/c mice, we firstly tested the effects of vaccines in a model consisting in the inoculation of stationary-phase promastigotes into the footpad. We observed that the disease evolution of the LieIF2 vaccinated mice was slower than in the control groups showing a significant decrease in footpad swelling in the last 3 weeks of the assay (Figure 9A). In addition, LieIF2 vaccinated mice showed a significant reduction of the parasite burdens in the popliteal LN draining the infected footpad with respect the burdens found in both control groups (Figure 9B). Also, the parasite dispersion to internal organs, measured as parasite load in the spleen, was very low in the LieIF2 vaccinated mice in relation to that found for the rest of the groups (Figure 9B). No significant differences in disease evolution or parasite burdens were observed between mice of the LieIF2B group and mice from both control groups (Figures 9A,B).

We also analyzed the evolution of cutaneous lesions in control and vaccinated animals after a challenge with highly infective metacyclic promastigotes in the ear dermis of mice from the BALB/c and C57BL/6 strains. In the BALB/c mice, LieIF2 and LieIF2B vaccinated animals showed a slower evolution of the lesions than the control groups that was more marked in the LieIF2 group (Figure 9C). However, in any case, lesion development was not contained and at the end of the study the size of the lesions was similar in mice from vaccinated or control groups. Local parasite loads mirrored lesion development. At week 5 post-challenge, LieIF2 and LieIF2B vaccinated animals showed a significant reduction of the parasite burdens in the ears with respect to both control groups (Figure 9D). Regarding the LN cells, only LieIF2 vaccinated mice showed lower number of parasites than control groups (Figure 9D). However, as occurred with the lesion size, at the end of the assay (8th week after challenge) similar parasite loads were observed at the ears and retromandibular LNs (Figure 9D). In addition, LieIF2 and LieIF2B vaccinated animals present a remarkable capacity to control *Leishmania* dispersion to internal organs as revealed by the low number of parasites detected in their spleens (Figure 9D). A partially protective effect of vaccination was also observed in vaccinated C57BL/6 mice. Both LieIF2 and LieIF2B vaccinated mice developed lower ear lesions after *L. major* inoculation than mice of control groups (Figure 9E). Control in lesion development was correlated to the presence of lower number of parasites in the ear and retromandibular LNs 5 weeks after challenge (Figure 9F, 5th week). At week 9 post-challenge animals from the control groups still showed higher parasite numbers than vaccinated animals (Figure 9F, 9th week).

### Analysis of the implication of LieIF2 and LieIF2B in the induction of IL-10 mediated responses in naïve mice

**Figure 10:**
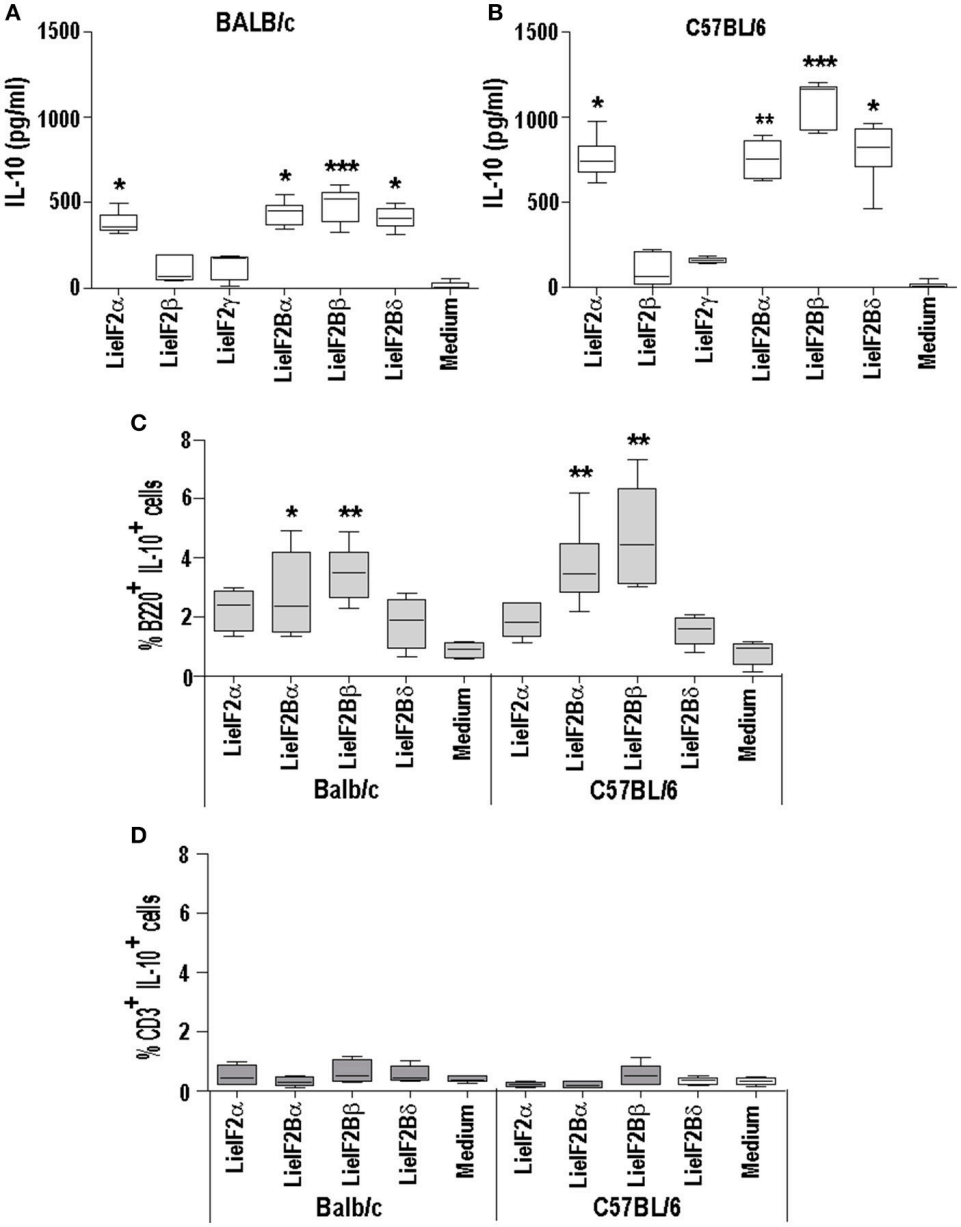
LieF2α, LieF2B β, and LieIF2δ subunits induce the secretion of IL-10 in B lymphocytes. Spleen cell cultures were established from naïve BALB/c or C57BL/6 mice (*n* = 6 each strain). BALB/c **(A)** of C57BL/6 **(B)** cells were independently cultured without stimulus (Medium) or with the indicated recombinant subunits. Graphs show the level of IL-10 culture supernatants. Frequencies of B220^+^ cells **(C)** of CD3^+^ cells **(D)** producing IL-10 in the cultures after recall with the indicated recombinant factors. Data are represented as Whisker (min to max) plots. Asterisks show the statistical differences between the data of the stimulated and the non-stimulated cultures (Kruskal–Wallis test).

As it is shown in Figures 5, 6, stimulation of spleen cells from either BALB/c or C57BL/6 control mice, receiving saline (or empty pcDNA) in the immunization schedule, with a mixture of LieIF2 (Figures 5A, 6A) or a mixture of LieIF2B (Figures 5B, 6B) subunits induced the production of moderate levels of IL-10. To obtain more details about the subunits(s) responsible of this effect, we processed the spleens from naïve mice (*n* = 6) and the splenocytes were grown in the absence or in the individual presence of the six subunits, monitoring the levels of IL-10 cytokine in the culture supernatants. The highest levels of IL-10 were found after stimulation with the LieIF2α subunit, and the three LieIF2B subunits in both BALB/c (Figure 10A) or C57BL/6 (Figure 10B) mice strains.

In a preliminary assay designed to analyze which cells were responsible for the production of IL-10, parallel cultures stimulated with these subunits, were analyzed by FACS to study the percentages of IL-10^+^ B cells (B220^+^) or IL-10^+^ T cells (CD3^+^). As it is shown in the Figure 10C, cultures stimulated with the four subunits showed higher percentages of B220^+^IL-10^+^ cells than the control unstimulated cultures, although statistical significance was only attained in the cultures stimulated with either LieIF2Bα or LieIF2Bβ subunits in both mouse strains; Figure 10C shows the data and statistics and Supplementary Figure 5A shows representative dot-plots. On the other hand, no differences were found for CD3^+^IL10^+^ cells between stimulated and unstimulated cultures (Figure 10D; Supplementary Figure 5B). As an additional control, stimulation of the splenocytes was carried out in the presence of amounts of LPS similar (0.1 ng/ml) and up to **two** orders of magnitude higher than those found in the protein preparations. In these conditions, we detected low levels of the cytokine in the supernatants (Supplementary Figure 5C). Moreover, no differences were found in the percentages of B220^+^IL10^+^ cells between LPS stimulated or non-stimulated cultures (Supplementary Figure 5D).

## Discussion

It is well-established that after *Leishmania* infection several intracellular parasite proteins interact with the immune system of the mammalian host. The existence of significant sequence divergence for many intracellular conserved protein families among *Leishmania* parasites and other eukaryotes is a common feature that may be due to the ancient position of *Leishmania* genus in the eukaryote phylogenetic tree (Sogin et al., 1986). From an immunological point of view, the existence of these differences is an important issue, since many of these protein families are antigenic in human and canine VL patients and the humoral and cellular responses are specific for parasite proteins without cross-reactivity with the host counterparts (Soto et al., 1999; Requena et al., 2000a; Maalej et al., 2003; Chenik et al., 2006). The similarity values obtained for the LieIF2 and LieIF2B subunits and their human orthologs range from 25 to 54% (Table 1). These values were comparable to those reported for other members of *Leishmania* translational machinery already characterized: *L. major* eIF3 factor subunits (20–25%, Rezende et al., 2014), *Leishmania donovani* eIF5A (45%, Singh et al., 2014) or *L. major* eIF4F subunits, (eIF4E, 22%; eIF4A, 56% and eIF4G, 25%, Dhalia et al., 2005). The data presented in this work demonstrate that the LieIF2 and LieIF2B are humoral markers of VL, since all the subunits were recognized by the sera of human and canine patients. The variability observed in the reactivity values found for each individual recombinant subunit in both mammalian hosts suggests the existence of individual differences in antigen recognition among human and canine patients. A similar behavior was observed in other studies performed with individual parasite antigenic proteins, assayed with sera collections obtained from patients naturally infected with the parasite in endemic areas (Soto et al., 1999; Maalej et al., 2003; Goto et al., 2009). Comparison of the percentages of positive individuals revealed important differences in the pattern of recognition between both species (Table 2). In humans, the LieIF2α subunit was the antigen recognized by a larger number of sera, whereas LieIF2Bβ and LieIF2γ were the most recognized by canine samples. Differences in canine and human immune responses to these and other *Leishmania* antigens may be reflecting differences in how the parasites interact with the immune system of both hosts (Goto et al., 2009).

On the other hand, when an experimental model of VL was employed, namely hamsters infected with *L. infantum*, we observed a 100% of positivity (Table 2). The high percentage value of antigenicity observed can be taken as an indication that the degree of individual variability in the recognition of both factors in this inbred experimental model is lower than that existing in human and dog natural populations. However, individual differences in the reactivity values of the hamster sera persisted, since data followed non-parametric distributions (Supplementary Figure 3C). Something similar occurred when the sera samples employed in this work were assayed with other antigenic proteins such as the surface protein KMP-11 or intracellular antigens such as PUF proteins, the HSP20, HSP70, and HSP83 stress proteins, the H2A and H3 histones or the LiP2a and LiP2b acidic ribosomal proteins (Requena et al., 2000b; Montalvo-Alvarez et al., 2008; Folgueira et al., 2010). Inclusion of this experimental model in our work allowed us to make a longitudinal analysis of the humoral response appearance. Interestingly, similar profiles were observed for the anti-LieIF2 or anti-LieIF2B response and antibody generation against SLA extracts, representing the whole parasite antigenic repertoire. A continuous increase in the intensity of the response against the factors and SLA found in this work (Figure 1) was concomitant to the increase in the number of proteins recognized by the sera of the hamster as shown previously (Requena et al., 2000b). At the end of the study, most of the parasite proteins became antigenic, being this observation in accordance with the induction of exacerbated humoral responses during disease progression in symptomatic VL human and canine patients (Miles et al., 2005; Kumar and Nylén, 2012; Fernandez-Cotrina et al., 2013; Hasker et al., 2014). It can be concluded then that the production of antibodies against both factors occurs from the first moments of infection. This demonstrates an early encounter between both factors and the immunological system of the host and it is ruled out that its antigenicity is due to the induction of the polyclonal responses associated with the pathology of the VL (Deak et al., 2010).

The intracellular location of the translation factors does not seem to be an impediment for their antigenicity. Similarly, many proteins with nuclear and cytosolic location, such as protein related to the translational machinery, enzymes implicated in parasite metabolism, heat shock proteins or histones have been described as immunodominant antigens not only in human and canine VL (Requena et al., 2000a; Coelho et al., 2009; Soto et al., 2009, 2015; Ramírez et al., 2013; Baharia et al., 2014; Sundar and Singh, 2014; Siripattanapipong et al., 2017), but also in human CL patients having moderate anti-*Leishmania* humoral responses (Ramírez et al., 2013; Souza et al., 2013; Duarte et al., 2015). Interaction of these proteins with B lymphocytes for antibody secretion may occur through complement mediated lysis of the non-metacyclic parasites causing the release of the whole internal cellular compounds (Mosser and Edelson, 1984; Ambrosio and De Messias-Reason, 2005; Moreno et al., 2010). Also, non-infective promastigotes can be lysed through the activity of neutrophil extracellular traps although metacyclic promastigotes are resistant to this innate immunity mechanism (Guimaraes-Costa et al., 2009, 2014; Hurrell et al., 2016). The release of different intracellular antigenic proteins contained in secreted vesicles (Silverman et al., 2008; Cuervo et al., 2009; Torrecilhas et al., 2012) may be also an alternative form of presentation of those antigens to the host immune system, since *Leishmania* is able to release microvesicles in the mammalian host (Silverman and Reiner, 2012) and their contents have been found to induce humoral responses in murine susceptible hosts (Hernández-Chinea, 2007), canine (Lima et al., 2016), and human patients (Soares et al., 2015) as well as inhibitory signaling for dendritic cells activation (Markikou-Ouni et al., 2015; Iborra et al., 2016; von Stebut and Tenzer, 2018). Interestingly, whereas in patients with the active form of the disease they induce strong humoral responses (Requena et al., 2000a; Maalej et al., 2003; Rafati et al., 2007; Costa et al., 2012; Souza et al., 2013) as well as IL-10 mediated responses (Bottrel et al., 2001; de Carvalho et al., 2003; Antonelli et al., 2004; Carvalho et al., 2005) in asymptomatic or in cured patients these proteins usually induce Th1-like responses (Probst et al., 2001; Bourreau et al., 2003; Baharia et al., 2014; Jaiswal et al., 2014; Cecilio et al., 2017). For this reason, these proteins are considered adequate humoral and cellular markers of infection, and the immune response elicited against them can be useful employed to monitor the development of the infection and also the success of the treatments.

A limitation of this work is that we have not tested cellular samples from natural leishmaniasis patients to analyze the cellular responses elicited against both factors. However, as an alternative, we moved onto murine models of leishmaniasis in order to understand the relationships of the parasite LieIF2 and LieIF2B and the host cellular immune system. First we found that these factors are also antigenic in mice infected with *L. infantum* (BALB/c) or *L. major* (BALB/c and C57BL/6) (Table 3). As it is deduced from data included in the Figure 2 and Supplementary Figure 4, the quality of the anti-LieIF2 or anti-LieIF2B humoral response was qualitatively similar to that observed for SLA, although with a lower intensity. The humoral response against the translation factors in the *L. infantum* infected BALB/c showed the mixed IgG1/IgG2a pattern found in this VL model against SLA, concomitant to the chronic infection in the spleen and the active destruction in the liver conducted by a parasite-specific T-cell dependent macrophage activation (Engwerda and Kaye, 2000; Garg and Dube, 2006; Loría-Cervera and Andrade-Narváez, 2014; Sacks and Melby, 2015). In the highly susceptible BALB/c-*L. major* model we found a predominant IgG1 antibody response against both factors, associated with the SLA-dependent Th2 response typically found in this mouse strain. In the resistant model C57BL/6-*L. major*, the low reactivity found against the factors was associated with the induction of IgG2c antibodies related to the generation of protective Th1-mediated responses (Sacks and Noben-Trauth, 2002; Sacks and Melby, 2015). The patterns of LieIF2- and LieIF2B-driven production of IFN-γ fits well with the different evolution of the disease in the three distinct murine models. Although in all cases production of this inflammatory cytokine is limited, there is a greater tendency for its secretion by cells from the C57BL/6 mice resistant to CL and in the BALB/c VL, where parasites are eliminated in the liver (Figure 3). On the other hand, the *L. major* infected susceptible animals were unable to produce LieIF2 and LieIF2B-dependent IFN-γ (Figure 3). Of note, a predominance of LieIF2 and LieIF2B factors-mediated IL-10 production was observed in the three experimental models of murine leishmaniasis regardless clinical evolution (Figure 3). This down-regulatory cytokine has been related to disease progression in mice models of CL (Noben-Trauth et al., 2003; Ronet et al., 2010; Buxbaum, 2015; Lee et al., 2017) or VL (Murphy et al., 2001; Faleiro et al., 2016) and in human CL or VL patients (Nylén et al., 2007; Carvalho et al., 2012, 2015; Gollob et al., 2014; Nabavi et al., 2018). Parasite proteins implicated in IL-10 production are being considered virulence factors and markers of disease. This is the case of the parasite KMP-11, a surface located protein that is implicated in the stimulation of IL-10 production by patients affected by CL and also in cultured murine macrophages infected *in vitro* by *L. amazonensis* (de Carvalho et al., 2003; de Mendonça et al., 2015). Similarly, the recombinant version of the papLe22 antigen is able to induce IL-10 secretion in human patients affected by VL (Suffia et al., 2000). In this work, we have found that the LieIF2- and LieIF2B-related IL-10 production occurred in the three murine models tested, although was higher in magnitude in the CL models (Figure 3). The immunological consequences of this production may be different. It has been previously reported that deficiency in IL-10 does not alter the final healing phenotype after *L. major* challenge in C57BL/6 mice (Schwarz et al., 2013). On the other hand, IL-10 plays a major role in CL disease evolution of BALB/c mice, as demonstrated by the healing phenotype shown by IL-10 deficient mice (Schwarz et al., 2013) or in the murine VL disease (Murphy et al., 2001). The fact that some of the subunits of the LieIF2 and LieIF2B factors are able to induce the secretion of IL-10 in spleen cells from both BALB/c or C57BL/6 naïve mice could be reinforcing the idea that these factors can be considered virulence factors. As mentioned above, the generation of early humoral responses to them is evidence of their rapid presentation to host B lymphocytes, cells that are also involved in the development of the disease throughout IL-10 production (Andreani et al., 2015; Silva-Barrios et al., 2017). The ability of the factors to induce the secretion of IL-10 in these cells would contribute to generate an anti-inflammatory environment in the infected tissues that would facilitate the progression of the parasite. A similar comportment has been postulated for the parasite cytosolic tryparedoxin, since it has been implicated in the induction of IL-10 by B cells of naïve mice (Cabral et al., 2008). The exposure of these intracellular *Leishmania* proteins may participate in immune-pathological processes by targeting B-cell to produce specific antibodies and leading to IL-10 secretion. The effect may be similar to the exposure to sand fly saliva factors that, by up-modulating IL-10 production enhance *Leishmania* infection in mice infected with cutaneous-tropic species (Norsworthy et al., 2004) or in human patients (Carvalho et al., 2012, 2015; Gollob et al., 2014). Data obtained in this work reinforce the implication of different parasite proteins in the modulation of the host immune system in order to facilitate the progress of infection. Previously reported examples are the LACK protein, a homolog of the receptor for activated C kinase in mammalian cells, which mounts an early IL-4 response after *Leishmania* infection (Launois et al., 1997) or some parasite secreted antigens that modulate C57BL/6 immune system toward a Th2 response (Tabatabaee et al., 2011). Also, the ribosomal protein S3a was found to be implicated in the induction of polyclonal expansion of B cells beside the inhibition of T cell proliferative responses (Cordeiro-Da-Silva et al., 2001).

Modulating the response against some of these immunologically active proteins by their administration in combination with adequate adjuvants was postulated as an interesting field of research for development of prophylactic or therapeutic vaccines (Badaro et al., 2001; Duarte et al., 2016b; Reguera et al., 2016). As a proof of concept, the inoculation of parasite ribosomal proteins combined with un-methylated CpG motives [ligands for the pro-inflammatory TLR-9 (Reed et al., 2013)] resulted in the protection against *L. major* infection inducing IFN-γ-mediated responses in C57BL/6 or BALB/c mice, correlated to the control of the humoral responses and IL-10 production driven by these parasite antigens in the last model (Iborra et al., 2008). In this work, as a proof of concept we tested DNA-vaccines based on both factors, taking advantage of the capacity of these genetic vaccines to induce IFN-γ mediated cellular responses specific for the proteins encoded in the plasmid vectors (Kaur et al., 2016; Kumar and Samant, 2016; Maspi et al., 2017). Our data showed that the LieIF2 and LieIF2B vaccinated mice produced IFN-γ in response to the corresponding subunits of both factors. However we also detected a LieIF2- and LieIF2B-specific production of IL-10 following immunization (Figures 5–8). In agreement with the results obtained in this work, the incapacity to control IL-4 or IL-10 production beside the induction of IFN-γ was considered as a bad marker for protection (Roberts et al., 2005) as it has been correlated with the inability to generate protective responses in BALB/c mice against *L. major* (Sjölander et al., 1998; Iborra et al., 2005, 2007) or *L. infantum* (Pirdel et al., 2014). The failure in protection showed in this work for the BALB/c CL model may be related to the necessity to control the responses mediated by IL-4 and by IL-10 besides generating IFN-γ as occurred with other tested vaccines against the parasite in CL (Gomes et al., 2012; Soto et al., 2015; Duarte et al., 2017) or VL models (Goto et al., 2011; Martins et al., 2017). On the other hand, protection in the C57BL/6-*L. major* model was associated to the induction of rapid IFN-γ mediated responses after infective challenge rather than the control of Th2 or IL-10 mediated responses as occurred with different vaccines based on parasite antigens or *Leishmania* live vaccines (Iborra et al., 2005; Kébaïer et al., 2006; Doroud et al., 2011; Peters et al., 2012; Solana et al., 2017). In this sense, the appearance of less severe lesions in C57BL/6 vaccinated mice after *L. major* infective challenge allows to reinforce the conclusion that both factors play a prominent role in the immune response after *Leishmania* infection.

We conclude that the subunits forming LieIF2 and LieIF2B factors are able to interact with the host immune system during *Leishmania* infection in different mammalian hosts. The induction of antibodies against the different subunits allows their classification as humoral markers of the disease. In addition, our findings related to the LieIF2 and LieIF2B production of IL-10 in mice infected with *L. major* also highlight the role of these factors as cellular markers of the disease and link them with the promotion of susceptibility against leishmaniasis. Since some of the LieIF2 and LieIF2B subunits are able to induce the secretion of IL-10 in B cells from naïve mice, they may be considered virulence factors implicated in the induction of early down-regulatory immune responses that may facilitate the progression of the infection. The induction of partial protective responses in C57BL/6 by the administration of LieIF2 or LieIF2B-based DNA vaccines opens the possibility of designing new formulations combining different subunits and adjuvants or new forms of antigen delivery to improve their prophylactic capacities.

## Author contributions

EG, SI, and MS: conceived and designed the experiments; EG, LC, JS, LR, and MS: performed the experiments; EG, LC, VG, MM, JR, SI, and MS: analyzed the data; CG-N, AB, MB-N, and JR: contributed reagents, materials, analysis tools; EG, JR, SI, and MS: wrote the manuscript. All authors read and approved the final version of the manuscript.

### Conflict of interest statement

The authors declare that the research was conducted in the absence of any commercial or financial relationships that could be construed as a potential conflict of interest. The handling Editor declared a past co-authorship with one of the authors JR.
