# Supplementary material for: Analysis of the Antigenic and Prophylactic Properties of the Leishmania Translation Initiation Factors eIF2 and eIF2B in Natural and Experimental Leishmaniasis
Source: Front Cell Infect Microbiol. 2018 Apr 5;8:112. doi: 10.3389/fcimb.2018.00112 (PMC5895769; doi:10.3389/fcimb.2018.00112)
Supplement: Supplementary file 1 [file Presentation1.pdf]

Supplementary Material

Analysis of the antigenic and prophylactic properties of the *Leishmania* translation initiation factors eIF2 and eIF2B in natural and experimental leishmaniasis.

Esther Garde, Laura Ramírez, Laura Corvo, José Carlos Solana, M. Elena Martín, Víctor M. González, Carlos Gómez-Nieto, Aldina Barral, Manoel Barral-Netto, José María Requena, Salvador Iborra*, Manuel Soto*.

*** Correspondence:** Corresponding Authors: SI salvador.iborra@cnic.es MS msoto@cbm.csic.es

1 Supplementary Figures and Tables.

1.1 Supplementary Tables

1.1.1 Supplementary Table 1

Primers employed for cloning the coding regions of the *Leishmania infantum* LieIF2 and LieIF2B subunits

Subunit	Forward primer (5'-3')	Reverse primer (5'-3')
**LieIF2α**	CGGGATCCATGGCTTCTTACTGTGTCACCG	CGGGATCCTTAGTCCGCATCCTCATCATC
**LieIF2β**	CGGGATCCATGATGCGCGAAGCCGGCCGCC	CGGGATCCTCACAGGATCATGGCGGCACG
**LieIF2γ**	CGGGATCCATGGCCGAGGAGGATATTGCG	CGGGATCCTTAGCTGCTCTGCTTCATGG
**LieIF2Bα**	CGGGATCCATGGACGTCCCAAGCGAAGA	CGGGATCCTTATCGCCGCTTTCCTGG
**LieIF2Bβ**	CGGGATCCATGCTGAGCAATGTGCCAGAAG	CGGGATCCTCAATCTGCGTCGCTGTAGTTC
**LieIF2Bδ**	CGGGATCCTCATCCCCGGACAAGATTCACG	CGGGATCCATGTCGTCGTCTTCGGAGAAG

Underlined are the *BamH*I restriction sites included for cloning purposes.

1.2 Supplementary Figures

1.2.1 Supplementary Figure 1

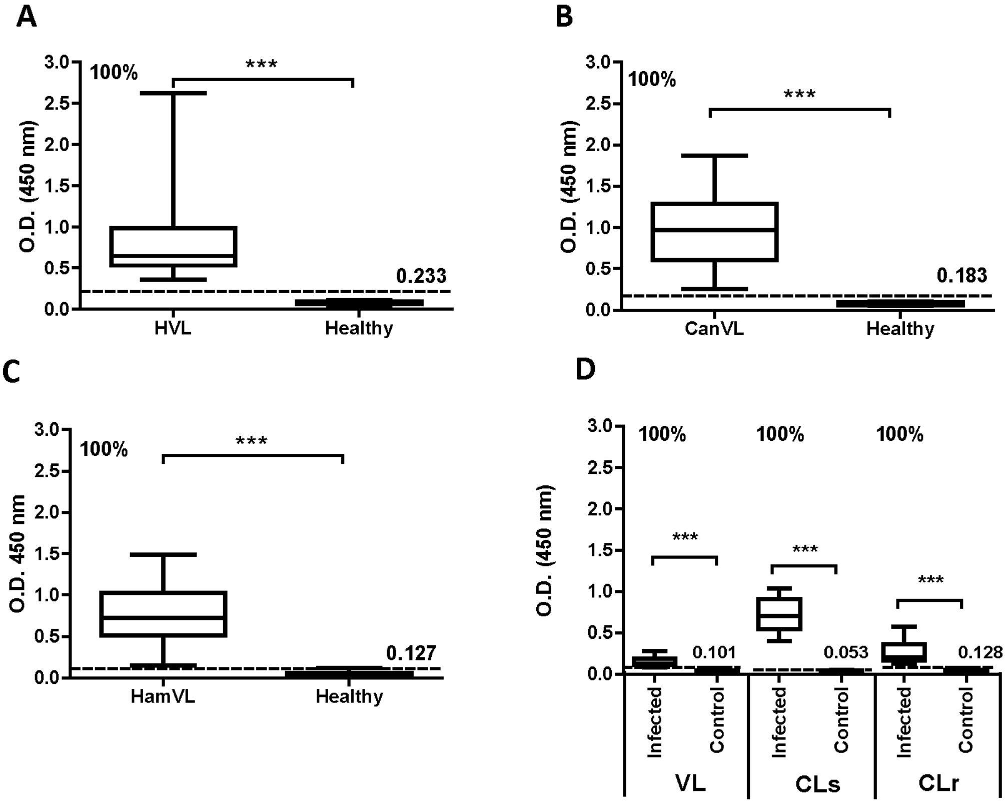

Supplementary Figure 1. Reactivity of the whole sera collection against Soluble *Leishmania* Antigen (SLA).

The next samples were employed: (A) Sera from human VL patients (HVL; n = 20) or healthy individuals (n = 10). (B) Sera from dogs naturally infected with *Leishmania infantum* (CanVL; n = 38) or sera from healthy dogs (n = 13). (C) Sera from hamster infected by the intracardiac route with *L. infantum* promastigotes (HamVL, 11 months after challenge; n = 9) or sera from healthy animals (n = 17). (D) Sera (n = 8 per group) from BALB/c mice infected intravenously with *L. infantum* (VL), BALB/c mice infected subcutaneously with *L. major* (CLs) or C57BL/6 mice infected intradermically with *L. major* (CLr). For each group sera collected previous to challenge was employed as healthy controls. Murine infected sera were taken at week 8 after challenge. Sera were individually tested for IgG reactivity by ELISA (1/200) against SLA. Horseradish peroxidase-conjugated anti-IgG antibodies (1/2000) for each specie were employed as the secondary reagent. Whisker (min to max) plots were employed to show the results. The percentage of positive sera is also indicated. Dotted lines represent the cut-off value for negative and positive samples, calculated by comparison of the reactivity values from infected and healthy groups using a Receiver Operating Characteristic (ROC) analysis and defined as the lowest O.D. value with a 100% of specificity. The cut-off value is indicated over the line. *** ($P < 0.001$) represent the statistical differences between infected and healthy groups evaluated by the nonparametric Mann-Whitney test.

1.2.2 Supplementary Figure 2

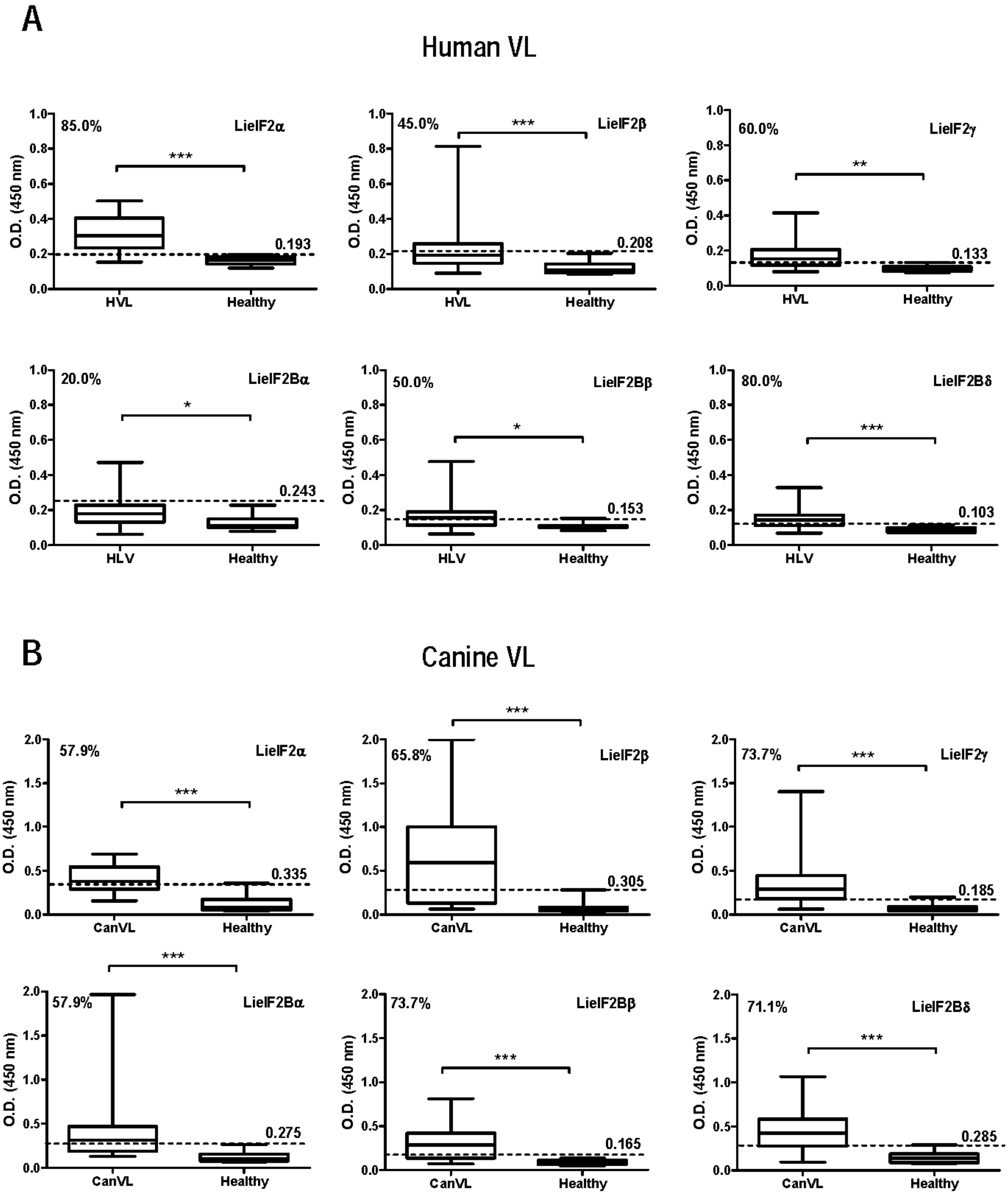

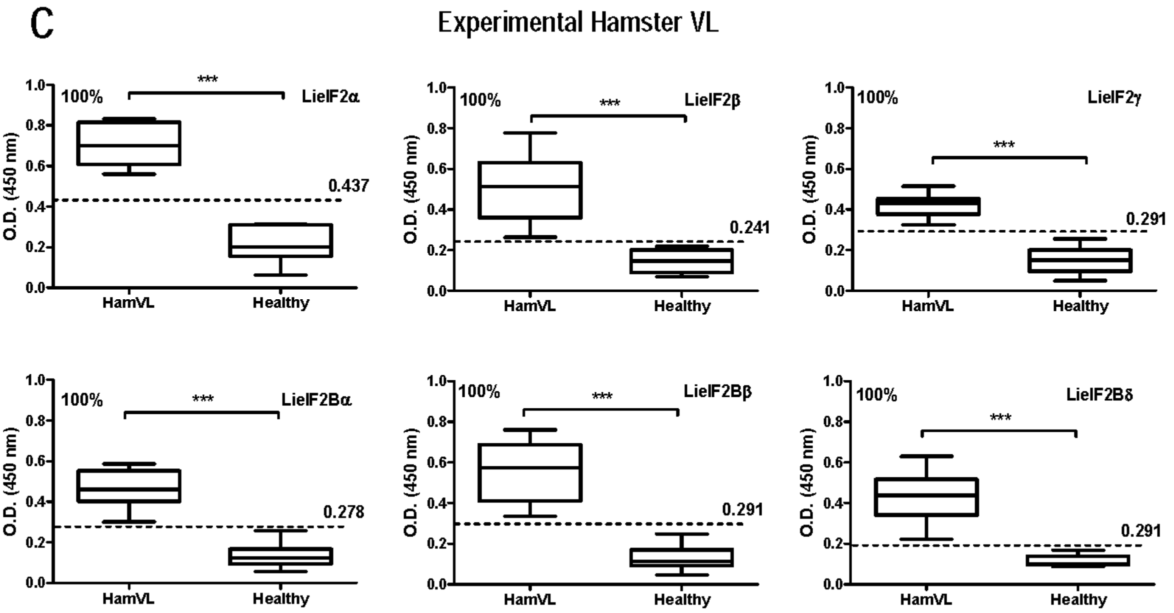

Supplementary Figure 2. Recognition of the *L. infantum* LieIF2 and LieF2B factors by sera from human or canine VL patients and hamster challenged with *L. infantum*. Human (A), canine (B) and hamster (C) sera collections described in Supplementary Figure 1 were individually tested for IgG reactivity by ELISA (1/200) against the three subunits of the LieIF2 factor (α, β and γ) or the LieIF2B factor (α, β and δ). Horseradish peroxidase-conjugated anti-IgG antibodies (1/2000) for each specie were employed as the secondary reagent. Whisker (min to max) plots are employed to show the results. The percentage of positive sera is indicated in each graph. Dotted lines represent the cut-off value for negative and positive samples, calculated by comparison of the reactivity values from infected and healthy groups using a Receiver Operating Characteristic (ROC) analysis and defined as the lowest O.D. value with a 100% of specificity. The cut-off value is indicated over the line. * ($P < 0.05$), ** ($P < 0.01$), *** ($P < 0.001$) indicate the statistical differences between VL and healthy groups (Mann-Whitney test).

1.2.3 Supplementary Figure 3

A

VL model (BALB/c-*L. infantum)*

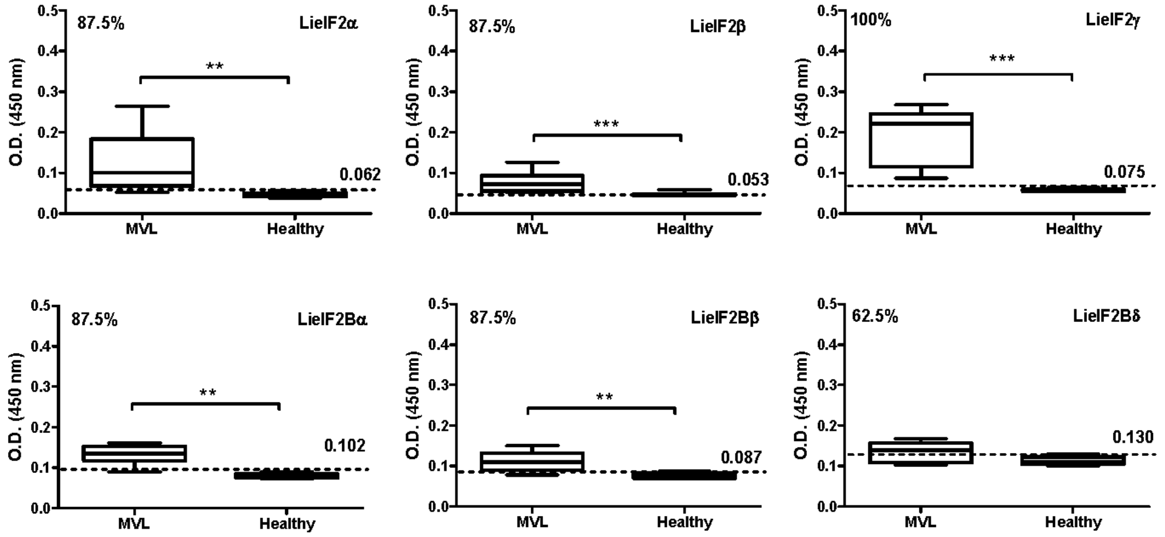

B

CL susceptible model (BALB/c-*L. major*)

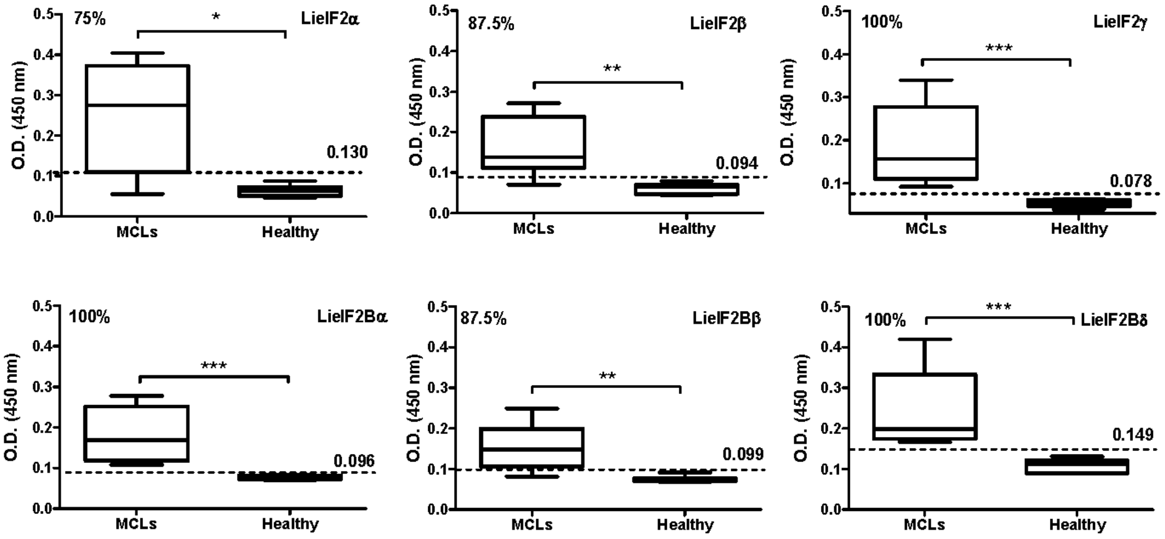

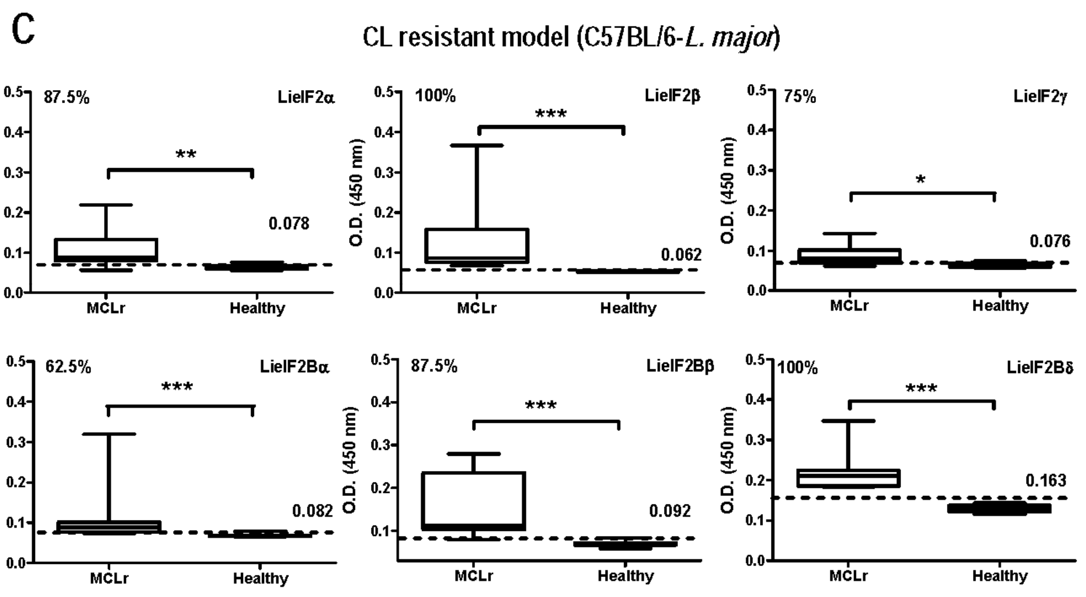

Supplementary Figure 3. Humoral response against the LieIF2 and LieIF2B proteins elicited in mice experimentally infected by *Leishmania.*

Sera from BALB/c mice infected with *L. infantum* (A) or *L. major* (B) and C57BL/6 mice infected with *L. major* (C) described in Supplementary Figure 1. were individually tested for IgG reactivity by ELISA (1/200) against the three subunits of the LieIF2 factor (α, β and γ) or the LieIF2B factor (α, β and δ). Horseradish peroxidase-conjugated anti-mouse IgG antibodies (1/2000) were employed as the secondary reagent. Whisker (min to max) plots are employed to show the results. The percentage of positive sera is indicated in each graph. Dotted lines represent the cut-off value for negative and positive samples, calculated by comparison of the reactivity values from infected and healthy groups using a Receiver Operating Characteristic (ROC) analysis and defined as the lowest O.D. value with a 100% of specificity. The cut-off value is indicated over the line. * ($P < 0.05$), ** ($P < 0.01$), *** ($P < 0.001$) indicate the statistical differences between VL and healthy groups (Mann-Whitney test).

1.2.4 Supplementary Figure 4

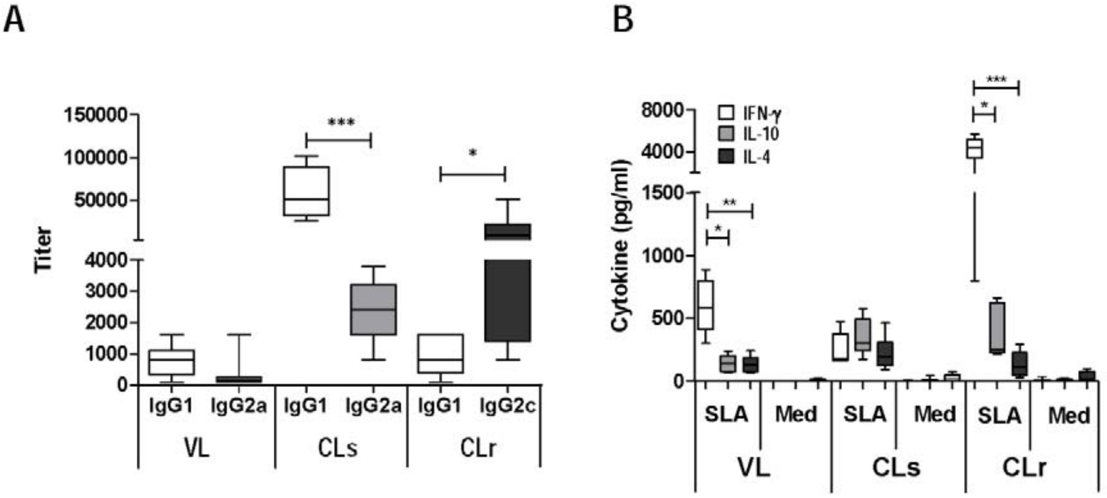

Supplementary Figure 4. Analysis of the SLA-dependent immune response in *Leishmania* infected mice.

Sera from mice infected as indicated in Supplementary Figure 3 were employed in ELISA to determine the anti-SLA IgG1 (all groups), IgG2a (BALB/c mice) or IgG2c (C57BL/6) titers by ELISA. Whisker (min to max) plots are employed to show the results. * ($P < 0.05$), *** ($P < 0.001$) indicate the statistical differences between the indicated subclasses titers (Mann-Whitney test) (A). Spleen cell cultures were established from *Leishmania* infected mice 8 weeks after challenge. Cells were independently cultured without stimulus (Med) or with SLA (12 µg/ml) for 72 h at 37°C, 5% CO_2. Whisker (min to max) plots are employed to show the levels of IFN-γ, IL-10 and IL-4 in culture supernatants measured by sandwich ELISA determined in 8 individual mice per group. * ($P < 0.05$), ** ($P < 0.01$), mark the statistical differences between cytokines (Kruskal-Wallis test) (B).

1.2.2 Supplementary Figure 5

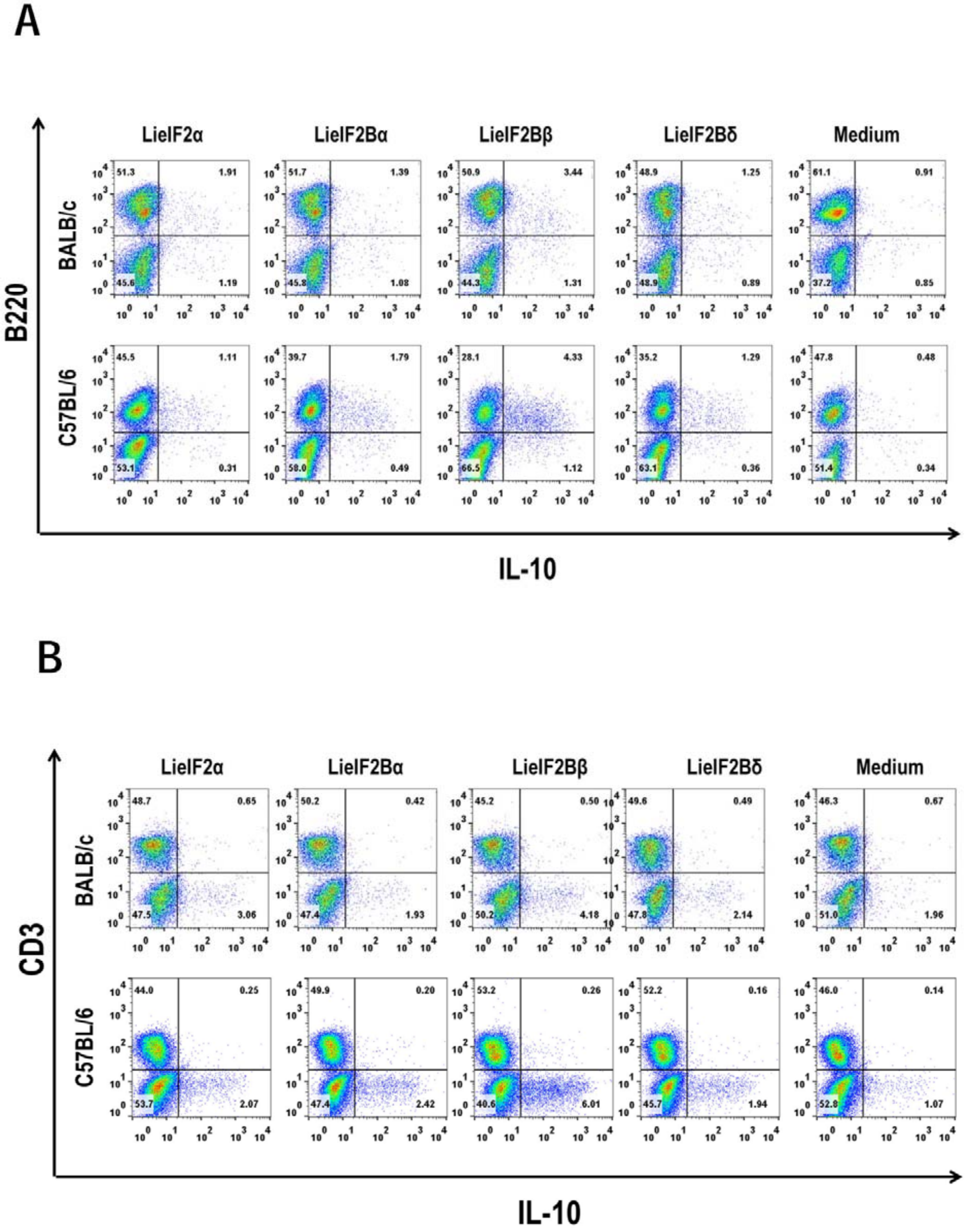

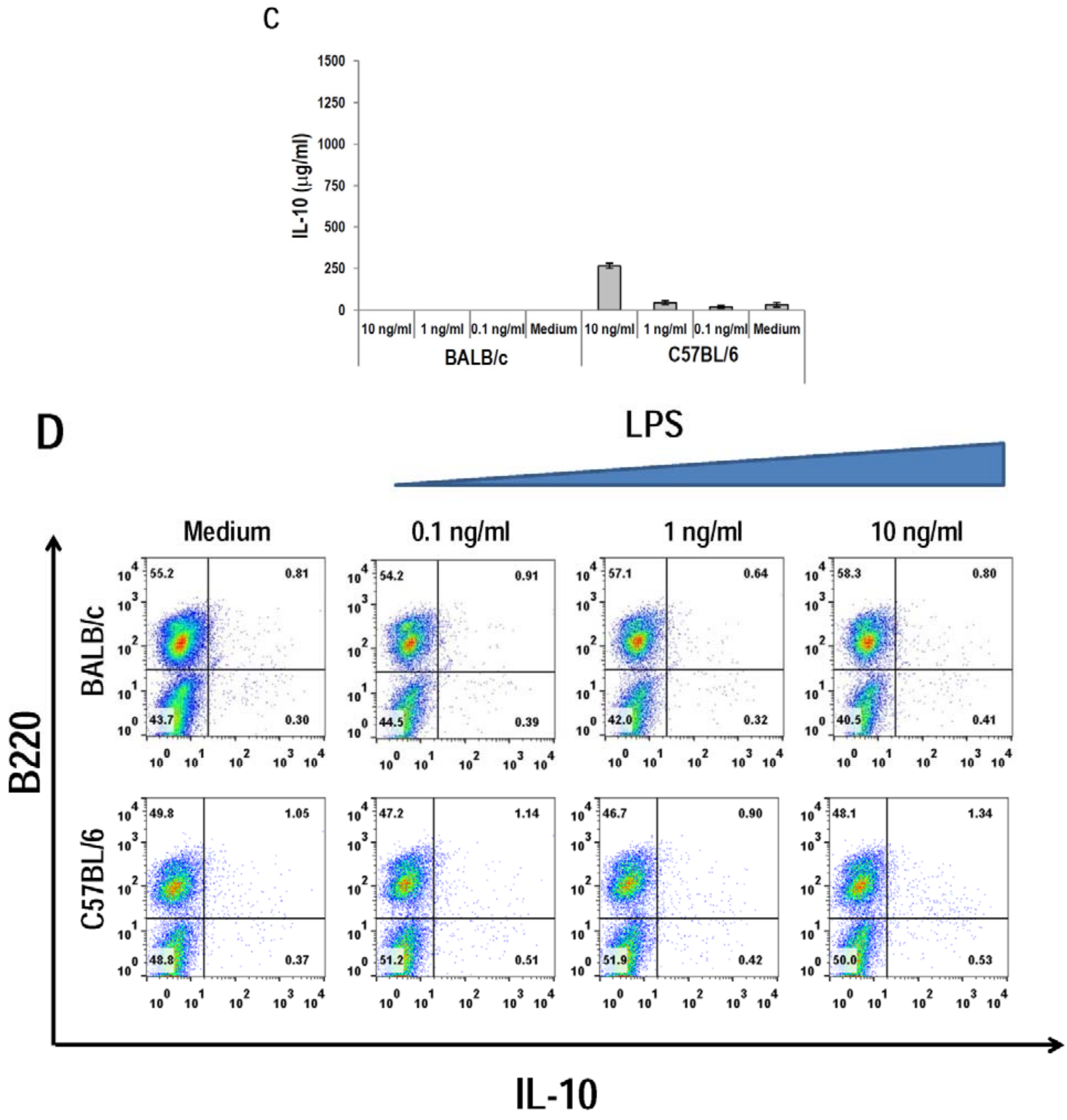

Supplementary Figure 5. Analysis of cytokine responses in naïve mice.

Representative dot plots of spleen cell cultures established from naïve BALB/c or C57BL/6 mice. Cells were independently cultured without stimulus (Medium) or treated with the recombinant LieIF2α, LieIF2Bα, LieIF2Bβ or LieIF2Bδ subunits and stained for intracellular IL-10 in B220+ (A) or CD3+ (B) cells. The amount of IL-10 was determined in the supernatant of cell cultures established from naïve BALB/c or C57BL/6 mice (pooled from 6 mice each strain) and independently cultured without stimulus (Medium) or with *E. coli* LPS (0.1 ng/ml, 1 ng/ml or 10 ng/ml) (C). Data are represented as mean ± SD. Representative dot plots of B220+ cells stained for intracellular IL-10 in LPS stimulated cultures described in C (D).
